# Stress drives the hippocampus to prioritize statistical prediction over episodic encoding

**DOI:** 10.1098/rstb.2025.0238

**Published:** 2026-07-09

**Authors:** Irene Zhou, Zihan Bai, Yuye Huang, Elaine G. Wijaya, Brynn E. Sherman, Nicholas B. Turk-Browne, Elizabeth V. Goldfarb

**Affiliations:** 1 Department of Psychology, Yale University, New Haven, CT, USA; 2 Wu Tsai Institute, Yale University, New Haven, CT, USA; 3 Department of Psychiatry, Yale University, New Haven, CT, USA; 4 Department of Psychology, New York University, New York, NY, USA; 5 Department of Psychological & Brain Science, Johns Hopkins University, Baltimore, MD, USA; 6 School of Medicine, UC Riverside, Riverside, CA, USA; 7 Department of Psychology, The Ohio State University, Columbus, OH, USA

**Keywords:** stress, hippocampus, statistical learning, behaviour, cognition, neuroscience

## Abstract

The hippocampus plays a critical role in encoding individual experiences into episodic memory, while also detecting patterns shared across these experiences that allow us to anticipate future events via statistical learning. Acute stress is known to impact the hippocampus but may have opposite effects on these competing functions. That is, although stress impairs episodic encoding, it may have different effects on statistical learning. Using functional magnetic resonance imaging, we tested how stress influences the hippocampal pathways and subfields that support episodic encoding and statistical learning while participants performed a task that engaged both processes. Across several analyses, stress biased hippocampal processing in favour of statistical learning, suppressing pattern separation between events in the dentate gyrus and enhancing the representation of statistically predictive features. Furthermore, stress drove the hippocampal monosynaptic pathway to support statistical learning rather than episodic encoding during predictive situations. Together, these data suggest that acute stress elicits targeted, adaptive changes in hippocampal pathways which may facilitate predicting and responding to upcoming events.

This article is part of the theme issue ‘The role of hippocampal predictions in cognition: bridging perception and memory’.

## 1. Introduction

Our lives contain many experiences that are unique but unfold in a reliable way. For example, a tree with a double-blaze marking on a hiking trail is often followed by a sharp turn in the trail. These reliable patterns can be detected automatically through statistical learning, a process which gradually extracts features that are shared across multiple experiences. Learning these regularities allows us to detect predictive situations and anticipate future events (looking for a hidden turn in advance). At the same time, unique details of individual experiences—such as a friend you encounter unexpectedly—are encoded rapidly and distinctively in episodic memory.

Despite episodic encoding and statistical learning operating on different timescales (fast versus slow, respectively) and prioritizing different features (unique versus shared, respectively), the hippocampus is thought to support both functions, albeit along distinct pathways. Neural network models suggest that statistical learning is supported by the monosynaptic pathway (MSP), which is a direct, recurrent connection between entorhinal cortex (EC) and hippocampal subfield CA1 that produces overlapping representations of related experiences over time. By contrast, episodic encoding is supported by the trisynaptic pathway (TSP), which connects EC to CA1 via intermediary connections through dentate gyrus (DG) and CA3 [1]. Functional neuroimaging supports this dual function, with hippocampal patterns supporting both predictive representations [2,3] and unique event signatures [4,5]. Although these learning processes are supported by different neural pathways, their shared dependence on CA1 as well as entorhinal inputs and outputs suggests that they may compete for shared neural resources that determine the representations and output of the hippocampus. Indeed, there are behavioural and neural trade-offs between making predictions and forming individual episodic memories [3].

The role of stress in arbitrating between these hippocampal functions is unknown. The hippocampus has long been known to be sensitive to acute stress [6–8]. Acute stress reduces plasticity [9] and elicits rapid dendritic atrophy [10,11] in rodent hippocampal neurons. In humans, acute stress reduces haemodynamic activity in the hippocampus and medial temporal lobe cortex [12,13] and alters hippocampal connectivity [14–16]. Such actions have been used to explain deleterious stress effects on episodic encoding [17,18], particularly for stimuli that are emotionally neutral and unrelated to the stressor [19]. However, recent studies suggest that statistical learning behaviour can be enhanced—rather than impaired—by stress [20,21], raising the possibility that stress exerts differential effects across hippocampal pathways.

A close examination of stress effects on subfields in the hippocampus suggests how stress might have opposite effects on episodic encoding and statistical learning. Across species, acute stress impairs adult hippocampal neurogenesis [22–24], which contributes to episodic encoding by supporting pattern separation—the formation of sparse, orthogonalized memory traces in DG to minimize interference [25–27]. Furthermore, acute stress and stress-related elevations in glucocorticoids suppress long-term potentiation inDG [28] and CA3 [29,30]. Notably, patterns differ for CA1, which has a lower density of glucocorticoid receptors [6,8]. Indeed, acute stress and glucocorticoids can spare or even enhance long-term potentiation in CA1 [29,31], facilitating neuronal firing in CA1 [32,33] but not in DG [34]. Together, these findings suggest that acute stress may impair subfields and connections involved in TSP, while sparing or enhancing those associated with MSP. However, the differential effects of acute stress on these hippocampal pathways in humans has not been tested.

The present study examines how acute stress affects hippocampal processes supporting episodic memory and statistical learning by combining functional magnetic resonance imaging (fMRI) with a task that assesses both functions simultaneously. We predict that stress impairs episodic encoding associated with TSP while sparing competing statistical learning processes supported by MSP. After either acute stress or a matched control procedure, participants viewed a stream of unique images structured into pairs of predictive and predictable scene categories [3]. The next day (to limit acute stress effects on retrieval [18,35]), participants completed behavioural tests assessing episodic memory (recognizing unique images) and retention of statistical learning (familiarity with pairs). We find that stress alters processing of predictive items across activity patterns within hippocampal subfields, as well as momentary connectivity along hippocampal pathways, with stress consistently prioritizing processing of statistically predictive information at the expense of episodic detail.

## 2. Methods

### (a) Participants

Participants were recruited from the New Haven community and pseudorandomly assigned to the stress or control groups to ensure balance in age, sex, and current subjective stress (Perceived Stress Scale (PSS) score). All participants were fluent in English, had a Body Mass Index between 18 and 35, and had normal or corrected-to-normal vision. To reduce factors that could influence stress-induced cortisol responses, participants were excluded if they were currently using psychiatric, *β*-blocker, or corticosteroid medications, or met criteria for any substance-use disorder. Female participants were not peri- or post-menopausal, pregnant, or lactating, and had not had a hysterectomy.

A total of 96 participants completed both experimental sessions (*n* = 49 stress (41% male, age 18–40, median age 23), *n* = 47 control (40% male, age 18–45, median age 24); sample size based on a power analysis from a prior study using a similar task (*d* = 0.42, 80% power [3])). Groups did not significantly differ in age (*t*(94) = 1.40, *p* = 0.166), sex (*χ*^2^(1, 96) = 0.0, *p* = 1.0), or PSS (*t*(94) = 0.69, *p* = 0.490). Of these participants, *n* = 4 stress and *n* = 4 control were excluded from fMRI analyses due to excess head motion, failure to complete the pre-learning and/or post-learning run, or inattentive responding (<60% of trials with response during stimulus presentation). One additional participant in the control group was excluded from fMRI analyses involving the learning runs because of a technical issue with fMRI data acquisition.

### (b) Study procedure

The experimental procedure is shown in figure 1A. Each participant completed two sessions on consecutive days. Before each session, they acclimated to the environment for at least 10 min (during which they completed the PSS [36]), allowing cortisol levels to stabilize [37].

On day 1, participants completed an encoding session during fMRI. During this session, participants were exposed to acute stress (*n* = 49) or a control manipulation (warm water bath; *n* = 47) [38]. After a 15 min delay [20], participants encoded sequences of unique scenes containing temporal regularities across three statistical learning runs. Before the stress induction, participants first completed a pre-learning scene task containing no temporal regularities, followed by a 6 min period of awake rest. After learning, participants again underwent a period of awake rest, followed by a post-learning run without regularities. Pupillometry data were collected during all scene-viewing runs as a non-invasive, online behavioural measure of sensitivity to statistical regularities.

On day 2, participants completed an item recognition test (assessing episodic memory) and a pair familiarity test (retention of statistical learning). All experimental sessions occurred between 12.00 and 18.00 to control for circadian fluctuations in cortisol [7].

#### (i) Stress induction and measurement

Participants in the stress group underwent the Socially Evaluated Cold Pressor Test (SECPT; [39]). Participants submerged their right arm in a bucket of ice water for 3 min (mean temperature = 2.79°C, s.d. = 1.02) under social evaluation, which involved being monitored by an experimenter with neutral affect wearing a laboratory coat while being recorded with a front-facing camera. Participants were told that the video recording would be used to evaluate their facial expressions and that they should look into the camera without speaking. Participants were not told how much time remained in the SECPT and were required to keep their arms submerged for the full 3 min. Participants in the control condition underwent a matched induction with warm water (mean temperature = 36.28°C, s.d. = 4.21) and no social evaluation (neither monitoring nor video recording).

Immediately before and after the stress/control procedure, participants completed the Positive and Negative Affect Schedule (PANAS) to assess changes in subjective affect. Participants also provided salivary samples throughout both visits by placing a Sarstedt salivette under their tongue for 2 mins (figure 1A). Samples were stored in a −20°C freezer in sterile tubes, then shipped frozen to the laboratory of Dr Andrea Gierens at the University of Trier for analysis of cortisol via time-resolved fluorescence immunoassay [40]. Because of insufficient sample, 23 of 384 samples from day 1 (from 15 participants, 6% of total) and 10 of 192 samples from day 2 (from nine participants, 5% of total) could not be assayed. A further three samples from day 1, and two samples from day 2 (all from the same participant) were excluded from analyses due to outlier cortisol levels (>4 s.d. above mean across groups).

#### (ii) Encoding task

The day 1 encoding task was based on Sherman & Turk-Browne [3] and Sherman *et al.* [20], and was conducted during fMRI. In each trial of the scene-viewing runs (pre-learning, learning, and post-learning), participants viewed a photograph of a scene (1 s) and judged whether the scene contained manmade objects using an MR-compatible button box (response mappings counterbalanced). Trials were separated by 2, 3.5, or 5 s intertrial fixations to jitter onsets for deconvolving event-related fMRI activity.

Each scene presented during encoding was trial-unique but drawn from one of 12 outdoor scene categories (beaches, bridges, canyons, deserts, fields, forests, lakes, lighthouses, marshes, mountains, parks, and waterfalls; figure 1B). Across the three statistical learning runs, scene categories were paired such that some categories (predictive; A, 3/12 categories) reliably preceded other categories (predictable; B, 3/12 categories) with a transition probability of 1.0 (figure 1B). Scenes from additional control (X, 6/12 categories) categories (neither predictive nor predictable) were interspersed randomly throughout the sequence (but never between an A–B pair), with assignment of categories to A/B/X conditions randomized per participant. The order of the image sequence within each statistical learning run was randomized with the following three constraints: category pairs and pairs of category pairs could not repeat back to back (i.e. no *A*_1_*B*_1_*A*_1_*B*_1_ or *A*_1_*B*_1_*A*_2_*B*_2_*A*_1_*B*_1_*A*_2_*B*_2_); repetitions of each category were spread equally across quartiles to minimize differences in study-test lag between categories; and the overall transition probability between ‘yes’ and ‘no’ responses on the manmade cover task was forced to be statistically indistinguishable from 0.5.

During pre- and post-learning runs, scenes from each category were presented individually in a random order, with no category pairs. These runs were used to obtain neural templates of category-level representations while avoiding active learning and temporal autocorrelation between A–B pairs. Participants were not informed of statistical regularities. Seven scenes from each scene category appeared in each scene-viewing run (84 scenes each in pre- and post-learning; 252 across learning runs).

#### (iii) Item recognition test

Episodic memory for each trial-unique scene encountered during learning was measured via an old/new recognition memory test on day 2. In each trial, participants were asked to judge whether the presented scene was ‘old’ (i.e. encountered on day 1) or ‘new’ (i.e. not seen on day 1; figure 1A, bottom left). After making an old/new response, participants rated their confidence (‘very unsure’, ‘unsure’, ‘sure’, ‘very sure’). All 252 scene images from the learning runs plus 252 foils (21 novel images from each category) were shown in a random order. Trials terminated following participant response or 6 s (whichever came earlier) and were followed by a 1 s intertrial fixation.

#### (iv) Pair familiarity test

Retention of statistically predictable scene categories was measured via a pair familiarity test. On each trial, a cartoon sketch of an A or B scene category (probe) was presented at the top of the screen (cartoons were used to avoid presenting a familiar or novel exemplar). Cartoons depicting two other scene categories—the paired category (match) and a category from the same condition in another pair (foil)—were presented at the bottom of the screen (e.g. if the probe is an A category, the match and foil are both B categories). Participants were asked to choose which category ‘goes with’ the probe (figure 1A, bottom right). Each probe/match/foil combination was tested once, resulting in four trials per category pair and 12 trials total. The positions of the match and foil categories were counterbalanced across trials. Each trial terminated following participant response or 6 s (whichever came earlier) followed by a 1 s intertrial fixation.

### (c) Data acquisition

#### (i) Pupil acquisition parameters

Pupillometry data were collected in a subset of participants (*n* = 33 stress, 28 control) because of technical constraints (primarily, the need for vision-corrective eyewear compatible with eye-tracking and for a clean line of sight to eye-tracking through the head coil). Pupil size was recorded continuously from one eye during scene-viewing runs on day 1 using an Eyelink 1000 infrared eye-tracker. Pupil size was recorded as the area of a model centroid in arbitrary units (AUs) specified by the eye-tracker’s firmware. Nine-point calibration and validation were performed each time the participant entered the scanner (immediately before the pre-learning run, and immediately before the first learning run) to ensure proper measurement of eye position.

#### (ii) fMRI acquisition parameters

Data were acquired at Yale University on a Siemens Prisma 3T MRI scanner with 64-channel head coil at the Brain Imaging Centre in the Faculty of Arts and Sciences (*n* with exclusions = 21 stress, 21 control) and at BrainWorks in the Centre for Neurocognition and Behaviour at the Wu Tsai Institute (*n* with exclusions = 24 stress, 22 control). Participants did not differ significantly across the two sites in age (*t*(86) = −1.26, *p* = 0.211) or condition assignment (*χ*^2^(1, 88) = 0.0, *p* = 1.0). Across all fMRI analyses, there were no significant effects or interactions with scanner site.

Functional images were acquired using an echo-planar imaging (EPI) sequence with the following parameters: repetition time (TR) = 1500 ms; echo time (TE) = 32 ms; 90 axial slices; voxel size = 1.5 mm isotropic; flip angle = 64°; multiband factor = 6. Anatomical scans included a T1-weighted magnetization prepared rapid gradient echo (MPRAGE) sequence (TR = 2400 ms; TE = 2.4 ms; voxel size = 1 × 1 ×1 mm; 208 sagittal slices; flip angle = 8°) and a T2-weighted turbo spin echo (TSE) sequence (TR = 11 170 ms; TE = 93 ms; 54 coronal slices; voxel size = 0.44 × 0.44 ×1.5 mm; distance factor = 20%; flip angle = 150°).

### (d) Data analysis

#### (i) Quantifying cortisol responses

Salivary cortisol levels were examined to assess physiological stress response following the SECPT. Due to skew, cortisol values were first normalized using a natural-log transform [41]. Differences in cortisol trajectory between groups were assessed using a linear mixed effects (LME) model (R 4.5.1, nlme package [42]) predicting log-transformed cortisol as a function of group, time-point, and their interaction, with participant as a random effect. Effect sizes for this and other linear mixed effects models were computed using the sjstats package [43]. We further summarized cortisol levels during learning (‘learning-concurrent cortisol’; mean (T3, T4) - T1; figure 1A). Participants with successful assays across T1, T3, and T4 were included in this analysis (*n* = 38 control, 43 stress).

#### (ii) Behavioural analyses

Performance on the item recognition test was summarized as hit rate, false alarm rate, and A′, a nonparametric measure of sensitivity [44]. Metrics were calculated separately for each scene condition (A, B, X). For the pair familiarity test, participant-level performance was summarized as the mean response accuracy overall, and pair-level performance was summarized as mean accuracy across the four trials presenting a given pair (separately per participant).

#### (iii) Pupil preprocessing

Pupil data were preprocessed in R (v. 4.5.1) using the GazeR package [45] following recommendations from previously established protocols [46]. A subset of 599 trials (2.92% of all trials) were excluded from analysis because they contained less than 50% usable raw datapoints during scene presentation; an additional 144 trials (0.70% of all trials) were excluded because of a lack of participant button press response to the cover task. Blinks and artefacts were detected using a velocity-based algorithm, and data were linearly interpolated during, 20 ms before, and 20 ms after blinks. Data were baseline-corrected at the trial level by subtracting the mean pupil size 0–50 ms after stimulus onset. Pupil size timeseries were then downsampled to 100 Hz.

#### (iv) Pupil analysis

Pupil size was used to examine differences in the online processing of different scene conditions during statistical learning runs. After preprocessing, the mean pupil size trace per condition from the pre-learning run was subtracted from that of the learning runs within participants (traces visualized in figure 2A). This ensured that condition-level differences in pupil size reflected learning. Data were then averaged per trial (across the 1 s stimulus presentation window) then per condition for each participant. Mean pupil sizes were compared between conditions using bootstrapping within-participant (see §2d(xi)). Group-level differences were assessed using LME to model mean pupil size as a function of group, condition, and their interaction, with participant as a random effect.

#### (v) fMRI preprocessing

fMRI data were preprocessed using FSL 6.0.3. A total of six runs from three participants (one control, two stress) were above the *a priori* motion threshold of <1.5 mm absolute mean frame-to-frame displacement as computed by FSL MCFLIRT [47]. These participants were excluded from all fMRI analyses. Data were skull-stripped, pre-whitened, and high-pass filtered at 0.01 Hz to remove low-frequency signal drift. FSL FEAT was then applied to regress out six linear estimated motion parameters and white matter timeseries plus their temporal derivatives as well as stick function regressors for nonlinear motion outliers. Residuals were aligned to the participant’s first functional scan. For all analyses, trial-evoked responses were normalized and time-shifted by 3 TRs (4.5 s) relative to task timing to account for haemodynamic lag. Trials with no participant response were excluded from all trial-level imaging analyses.

#### (vi) fMRI region of interest definitions

To examine neural representations and connectivity along hippocampal pathways, we segmented entorhinal cortex (EC) and hippocampal subfields CA1, CA2/3, and DG using the automatic segmentation of hippocampal subfields (ASHS) package [48] with the Princeton Young Adult 3T ASHS Atlas [49] from each participant’s T1- and T2-weighted anatomical scans. As we did not have lateralized predictions, region of interest (ROI) masks were combined across hemispheres. These participant-specific ROIs were then aligned into that participant’s functional space. For all scene-viewing runs, preprocessed data were masked to include only voxels within an ROI, then *z*-scored across time for subsequent analyses.

#### (vii) Predictive category decoding analysis

A multivoxel pattern classification approach was used to determine whether statistical learning led to neural evidence for predicted categories [3]. After preprocessing and extracting data from ROIs, voxel activity patterns from timepoints corresponding to stimulus presentation of B images were extracted from each participant’s pre-learning run as a training set for a classifier. For each participant, binary linear support vector machines (SVMs) were trained to distinguish B categories (e.g. *B*_1_ = forests, *B*_2_ = fields, and *B*_3_ = bridges) using data and labels from the pre-learning run. As each participant encountered three distinct A and B categories over the course of the experiment, three different binary classifiers were trained to distinguish between pairs of B categories (*B*_1_ versus *B*_2_, *B*_1_ versus *B*_3_, *B*_2_ versus *B*_3_). SVMs were defined using the SVC function in scikit-learn with a penalty parameter of 1.00.

These classifiers were then tested on neural data while participants viewed the corresponding A images in the post-learning run (e.g. a classifier trained to distinguish *B*_1_ versus *B*_2_ in pre-learning was tested on*A*_1_ (e.g. beach) versus *A*_2_ (mountain) trials in post-learning). The post-learning run was used to avoid temporal autocorrelation between paired A and B images in the learning runs (A was always followed by B); the training and testing sets were thus drawn from runs with randomized temporal structure. Classifier accuracy was defined as the percent of correct predictions (e.g. a classifier made a correct prediction if an *A*_1_ scene was classified as *B*_1_ over *B*_2_). Accuracy was averaged over all three classifiers, resulting in one mean accuracy score per participant. To assess reliability at the group level, performance was compared with an assumed chance level of 0.5 (given the binary nature of the classifier) across participants using bootstrapping.

To verify our results without assuming chance accuracy, we performed randomization tests in which we computed an empirical null distribution of classification accuracy values for each participant. Null distributions were generated from 9999 iterations of shuffling A category labels in each participant’s post-learning run before extracting binary testing sets to score each classifier. Scores were then averaged within participant to produce a mean (null) score for each iteration. We then converted each participant’s true classification accuracy to a *z*-score relative to their own null distribution, and tested reliability at the group level by comparing *z*-scored accuracies against 0 using bootstrapping.

Before training and testing each classifier, feature selection was performed by selecting a subset of voxels using the SelectKPercent function in Python’s scikit-learn module. The percentage of voxels selected from each ROI was consistent across all participants (CA1: 12.5%; CA2/3: 20%; DG: 12.5% of voxels). The best value for this percentage parameter was determined across all participants by using nested leave-one-out cross-validation within the training data only.

#### (viii) Category representation similarity analysis

A multivoxel pattern similarity approach was used to assess pattern separation during learning (see figure 3B). After preprocessing and extracting data from ROIs, voxel activity patterns from timepoints corresponding to stimulus presentation were extracted from each participant’s learning runs. For each trial, *within-category* pattern similarity was computed as the mean Pearson correlation between the activity pattern for that trial (e.g. a beach) and all other trials from the same scene category (all other beaches) during that learning run. *Between-category* pattern similarity was computed as the mean Pearson correlation between the activity pattern for that trial (e.g. a beach) and all other trials from different categories within the same condition (that is, all non-beach images from other A categories) during that learning run. After normalizing mean correlations using a Fisher *r*-to-*z* transform, between-category pattern similarity was subtracted from within-category pattern similarity, and the resulting values were averaged within participant and condition. Values less than or equal to 0 reflect pattern separation, indicating that scenes within the same category were represented more distinctly than scenes from different categories. Group-level evidence for pattern separation was assessed by comparing against a null value of 0 across participants using bootstrapping. Within-group differences between conditions were assessed using paired-samples bootstrapping, and within-condition comparisons across groups were assessed using between-groups bootstrapping.

#### (ix) Category evidence analysis

Multivoxel pattern similarity was also used to quantify category-level evidence in each learning trial (see figure 3C), or the mean Pearson correlation between the activity pattern for that trial and all trials with the same scene category from the pre-learning run (e.g. a beach from learning compared with every beach from pre-learning). Higher values indicated greater similarity between a particular item and the category overall, or more of a category-level representation. Resulting trialwise category evidence values were then normalized using a Fisher *r*-to-*z* transform then averaged within participant and condition. As in §2d(viii), comparisons against 0, within-group differences between conditions, and within-condition comparisons across groups were assessed using bootstrapping.

#### (x) Momentary cofluctuation analyses

In addition to examining patterns within subfields during learning, we also calculated functional coupling between subfields within hippocampal pathways. Given their overlapping role in both MSP and TSP, we focused on coupling between CA1 and EC.After preprocessing, we used a GLM to fit stimulus-evoked responses with a canonical double-gamma haemodynamic response function (plus temporal derivative) for each trial. We then extracted the residuals of this model (variance not explained by univariate responses) from the voxels in each subfield and calculated the mean time-series across voxels. Using an edge timeseries approach [50,51], we quantified momentary cofluctuation for pairs of subfields by *z*-scoring their residual timeseries and multi-plying them on a timepoint-by-timepoint basis. Averaging the resulting edge timeseries across time (more specifically, summing and dividing by the total number of timepoints minus 1) yields the Pearson correlation (i.e. background connectivity; see [52–54]) between subfields. Thus, timepoints with large positive products contribute to more positive overall connectivity, whereas those with large negative products contribute to more negative overall connectivity.

We then determined how connectivity between subfields during predictive images related to episodic encoding and statistical learning. For episodic encoding, we compared momentary average cofluctuation values between subsequently remembered and forgotten A images (that is, correctly identified as ‘old’ and incorrectly identified as ‘new’, respectively, in the item recognition test on day 2). For statistical learning, we related the average momentary cofluctuation for each predictive A category to behavioural familiarity for the corresponding A–B pair. To facilitate comparison with the episodic memory approach, we repeated the statistical learning analysis after binarizing performance on the pair familiarity test, labelling pairs with above-chance (>0.5) accuracy as ‘learned’ and pairs with below-chance (<0.5) accuracy as ‘not learned’, and omitting pairs with at-chance (= 0.5) accuracy. For the continuous version of this analysis, see electronic supplementary material, figure S6A.

#### (xi) Bootstrap statistical testing

Unless otherwise stated, statistical tests were conducted using a non-parametric bootstrap resampling method [55] with 99 999 iterations. For one-sample testing or paired-sample comparisons, participants were resampled with replacement and the group mean computed for each iteration. For between-participants comparisons, participants were resampled with replacement within groups, and the difference between group means was computed for each iteration. For comparisons against chance, bootstrapped *p*-values were calculated as the proportion of sampling iterations yielding a group mean less than or equal to chance. For two-tailed comparisons (e.g. between groups or conditions), the *p*-value was calculated as the proportion of sampling iterations yielding a difference in the opposite direction of the true difference, multiplied by 2. We report the rank-biserial correlation (*r*) as a measure of effect size for comparable non-parametric tests (Wilcoxon signed-rank test for one-sample and paired-sample tests, and Mann–Whitney rank-sum test for independent two-sample tests). For correlations, participants were resampled with replacement and the Pearson correlation between the two variables of interest was recalculated for each iteration. The resulting correlation coefficient (*r*) from each iteration was used to populate a sampling distribution. *P*-values were calculated as the proportion of sampling iterations in which the sampled correlation was the opposite sign from the true correlation, multiplied by 2 for two-tailed significance. In all cases, 95% confidence intervals were defined as the 2.5th and 97.5th percentiles of the sampling distribution.

## 3. Results

### (a) Stress manipulation evoked changes in subjective affect and cortisol

We first evaluated the efficacy of the stress induction by examining changes in subjective affect (PANAS scores; figure 1C). Participants in the stress group felt significantly more negative after the SECPT (95% CI: [0.88, 5.63], *p* = 0.009, *r* = 0.548), whereas participants in the control group reported feeling significantly *less* negative affect (95% CI: [−4.06, −0.80], *p* = 0.001, *r* = −0.549). Change in negative affect was significantly higher in the stress group than in the control group (95% CI: [2.74, 8.51], *p* < 0.001, *r* = 0.514).

Given the importance of glucocorticoids for stress effects on hippocampal function, we next assessed whether the stress induction increased salivary cortisol levels during learning. Based on previous work [20], we timed the onset of learning relative to SECPT with the expectation that cortisol would be elevated from the beginning to the end of learning. As anticipated, groups showed significantly different cortisol trajectories throughout day 1 (figure 1D; main effect of group: *F*(1, 79) = 1.58, *p* = 0.212, $\eta_{p}^{2}=0.006$; main effect of timepoint: *F*(1, 240) = 15.20, *p* < 0.001, $\eta_{p}^{2}=0.056$; group × timepoint: *F*(1, 240) = 74.26, *p* < 0.001, $\eta_{p}^{2}=0.226$). In the stress group, cortisol was significantly elevated during learning (average of pre- and post-learning minus base-line; 95% CI: [0.45, 0.85], *p* < 0.001, *r* = 0.850), whereas cortisol was significantly reduced during learning in the control group (95% CI: [−0.40, −0.10], *p* = 0.002, *r* = −0.549; stress versus control: 95% CI: [0.65, 1.14], *p* < 0.001, *r* = 0.739). These stress effects were specific to learning, as we did not observe any differences in cortisol between groups on the following day during the retrieval session (main effect of group: *F*(1, 84) = 0.25, *p* = 0.620, $\eta_{p}^{2}=0.002$; main effect of timepoint: *F*(1, 84) = 2.04, *p* = 0.157, $\eta_{p}^{2}=0.020$; group × timepoint: *F*(1, 84) = 1.14, *p* = 0.288, $\eta_{p}^{2}=0.011$). Across all subsequent analyses, main effects were not related to individual differences in learning-concurrent cortisol.

### (b) Both groups showed pupillary evidence of statistical learning

We next examined whether participants were sensitive to the regularities in the scene sequence during statistical learning. If participants learned the regularities, they should show differential responses to the A, B, and X scene conditions that were otherwise matched. We anticipated that these differences would be reflected in pupil responses. Indeed, there was a significant difference in mean pupil size between A and B, with the control group exhibiting more constriction (smaller pupil) during B (A versus B: 95% CI: [2.00, 20.28], *p* = 0.018, *r* = 0.483; A versus X: 95% CI: [−12.35, 13.49], *p* = 0.936, *r* = 0.020; B versus X: 95% CI: [−19.68, −1.62], *p* = 0.021, *r* = −0.468) and the stress group exhibiting more constriction during A (A versus B: 95% CI = [−31.36, −4.32], *p* = 0.008,*r* = −0.483; A versus X: 95% CI: [−21.28, −1.85], *p* = 0.018, *r* = −0.376; B versus X: 95% CI: [−4.74, 17.55], *p* = 0.273, *r* = 0.251). Although stress flipped the direction of these effects (group × condition: *F*(2, 118) = 6.12, *p* = 0.003, $\eta_{p}^{2}=0.088$), the observation that participants in both groups showed differential pupillary responses to predictive and predictable images suggests sensitivity to learned regularities.

### (c) Both groups showed neural evidence of prediction in CA2/3 after statistical learning

We expected that statistical learning would facilitate prediction of upcoming B categories while viewing paired A categories. Based on previous work [3], we anticipated that this learned prediction would be evident in hippocampus; within the hippocampus, such effects have been observed reliably in the CA2/3 subfield [2,56,57]. We thus tested whether expected B categories could be reliably decoded from patterns of activity in hippocampal subfields during A images *after* learning (using a classifier trained to separate B categories *before* learning; approach shown in figure 2B).

We found successful prediction in both groups. The upcoming B category could be reliably decoded during A images in CA2/3 for both control (95% CI: [0.50, 0.54], *p* = 0.036, *r* = 0.371) and stress (95% CI: [0.50, 0.55], *p* = 0.023, *r* = 0.382) groups. These effects were specific to CA2/3 and not present in CA1 (control 95% CI: [0.49, 0.53], *p* = 0.092, *r* = 0.289; stress 95% CI: [0.47, 0.51], *p* = 0.186, *r* = −0.144) or DG (control 95% CI: [0.47, 0.51], *p* = 0.243, *r* = −0.119; stress 95% CI: [0.49, 0.52], *p* = 0.275, *r* = 0.142). As chance classification can deviate from hypothetical levels, we confirmed these results using randomization tests (electronic supplementary material, figure S1). These predictive representations provide offline (i.e. post-learning) neural evidence that both groups were sensitive to statistical regularities, complementing the online evidence from pupillometry.

### (d) Acute stress impairs episodic memory for predictive items

Despite comparable indications of statistical learning, we did find differences in episodic encoding for participants exposed to stress. Participants in both groups had above-chance episodic memory for A, B, and X images the next day (A′ versus chance = 0.5; *p* < 0.001; figure 3A). Examining memory within groups, the control group had significantly better memory for A images (A versus X: 95% CI = [0.005, 0.052], *p* = 0.018, *r* = 0.346; B versus X: 95% CI = [−0.019, 0.035], *p* = 0.543, *r* = 0.126), a pattern that was not evident in the stress group (A versus X: 95% CI = [−0.031, 0.016], *p* = 0.509, *r* = −0.089; B versus X: 95% CI= [−0.051, 0.001], *p* = 0.063, *r* = −0.324). Considering memory between groups, episodic memory was notably impaired in the stress group relative to the control group for predictive A images (95% CI: [−0.061, −0.002], *p* = 0.035, *r* = −0.235), which was not evident for predictable B images (95% CI: [−0.063, 0.006], *p* = 0.109, *r* = −0.228) or baseline X images (95% CI: [−0.024, 0.033], *p* = 0.752, *r* = 0.048), although stress-induced impairments were not significantly stronger for A compared with other conditions (LME predicting A′ based on group and condition; main effect group: *F*(1, 94) = 2.45, *p* = 0.121, $\eta_{p}^{2}=0.011$; group × condition: *F*(2, 188) = 2.31, *p* = 0.102, $\eta_{p}^{2}=0.020$).

### (e) Acute stress impairs neural pattern separation of predictive items

We examined neural representations during encoding to understand how stress altered episodic memory. Within our task, predictive scenes—which showed significantly impaired episodic memory under stress—may invoke conflicting computations that compete for shared hippocampal resources. To form strong episodic memories, scenes need to be encoded as distinct events [58]. DG supports this process through pattern separation [4,5]; thus, we would expect DG patterns associated with a scene to be relatively *uncorrelated* with patterns for other scenes from the same category. On the other hand, statistical learning requires processing of commonalities between scenes from the same category to enable extraction of category-level scene transitions. In this case, we would expect patterns associated with a scene to be *more correlated* with other scenes from the same category.

To quantify these processes, we computed mean multivoxel pattern similarity during learning between BOLD activity patterns in DG for scenes from the same category relative to mean pattern similarity with scenes from different categories in the same condition (figure 3B) [59]. Consistent with pattern separation, this difference was not significantly greater than zero for any condition in the control group (A: 95% CI: [−3.18, 1.47] × 10^−3^, *p* = 0.757, *r* = −0.099; B: 95% CI: [−2.18, 2.43] × 10^−3^, *p* = 0.475, *r* = −0.037; X: 95% CI: [−2.84, −0.054] × 10^−3^, *p* = 0.979, *r* = −0.400), and for B and X conditions in the stress group (B: 95% CI: [−2.81, 2.21] × 10^−3^, *p* = 0.583, *r* = −0.009; X: 95% CI: [−1.16, 1.38] × 10^−3^, *p* = 0.426, *r* = 0.032).

By contrast, the stress group showed less pattern separation for predictive A scenes, with significantly higher within- than between-category similarity in DG (95% CI: [0.49, 4.02] × 10^−3^, *p* = 0.006, *r* = 0.391). This result differed significantly from how these same participants processed baseline X scenes (95% CI: [0.18, 4.12] × 10^−3^, *p* = 0.016, *r* = 0.333) and how the control group processed A scenes (95% CI: [0.19, 6.03] × 10^−3^, *p* = 0.018, *r* = 0.228). These effects were driven primarily by differences in within-category similarity (electronic supplementary material, figure S2), and were not present in hippocampal subfields CA1 or CA2/3 in any condition or group (electronic supplementary material, figure S3).

### (f) Hippocampus represents predictive categories under stress

Our results suggest that stress disrupts pattern separation among predictive scenes in DG, which may support processing category-level information useful for predicting the next category. To quantify category-level information directly, we calculated the mean multivoxel pattern similarity between each scene during learning and all scenes from the same category in the pre-learning run, which provides a ‘category template’ unaffected by temporal regularities (figure 3C).

The findings from this category evidence analysis mirror and extend the pattern separation results. For the control group, category evidence in DG was not significantly greater than zero in any condition (A: 95% CI: [−0.59, 1.04] × 10^−3^, *p* = 0.331, *r* = −0.034; B: 95% CI: [−1.25, 0.59] × 10^−3^, *p* = 0.736, *r* = −0.014; X: 95% CI: [−0.79, 0.66] × 10^−3^, *p* = 0.55, *r* = 0.061). However, the stress group showed significant DG category evidence for predictive A scenes (95% CI: [0.53, 2.16] × 10^−3^, *p* < 0.001, *r* = 0.420), but not for B (95% CI: [−0.93, 0.82] × 10^−3^, *p* = 0.558, *r* = −0.063) or X (95% CI: [−0.50, 0.72] × 10^−3^, *p* = 0.354, *r* = 0.071). Category evidence in the stress group for A was significantly stronger than for B (95% CI: [0.11, 2.73] × 10^−3^, *p* = 0.016, *r* = 0.302) or X (95% CI: [0.26, 2.20] × 10^−3^, *p* = 0.006, *r* = 0.407) within participants, as well as category evidence observed in the control group for A (95% CI: [−0.02, 2.30] × 10^−3^, *p* = 0.027, *r* = 0.225). There was a similar, though marginally significant, pattern of results in CA1 but not in CA2/3 (electronic supplementary material, figure S4).

These results indicate that, although DG does not typically represent scene categories, stress drives DG representations to code for this information, perhaps because it is relevant to prediction. To test whether category evidence is indeed beneficial to statistical learning, we examined whether A category evidence in DG during learning was associated with subsequent prediction of B in CA2/3 after learning (figure 2D). Indeed, stronger A category evidence positively correlated with prediction in both groups (stress: *r*(43) = 0.35, *p* = 0.024; control: *r*(40) = 0.29, *p* = 0.038). On the other hand, representing A scenes at a categorical level could impair encoding of idiosyncratic features necessary for episodic memory. Consistent with this idea, stronger A category evidence tracked more false alarms in the stress group (*r*(43) = 0.32, *p* = 0.040; figure 3E) but not the control group (*r*(40) = 0.019, *p* = 0.896). Together, these results suggest that stress leads the hippocampus to represent statistically predictive information at the expense of episodic memory precision.

### (g) Stress prioritizes statistical prediction in monosynaptic pathway

Beyond individual subfields, hippocampal circuit pathways arbitrate between statistical learning and episodic encoding. In particular, the direct connection between CA1-EC is common to both TSP (episodic encoding) and MSP (statistical learning); thus, activity along this pathway could potentially reflect a trade-off between these memory processes. If CA1-EC supports episodic encoding, we would expect differential cofluctuation for scenes that were subsequently remembered versus forgotten. On the other hand, if CA1-EC supports statistical learning, we would expect differential cofluctuation for scenes in pairs that were subsequently retained in the pair familiarity test. Here we test how stress influences the representation of statistical and episodic encoding within this overlapping pathway.

As stress primarily impacted hippocampal processing of predictive A items, we used an edge timeseries approach to isolate momentary cofluctuation between CA1 and EC during A scenes. Momentary cofluctuation in CA1-EC was related to both episodic encoding and statistical learning, but these relationships were modulated by stress (figure 4). In the control group, CA1-EC cofluctuation tracked episodic encoding, with higher cofluctuation during subsequently remembered compared with forgotten A items (figure 4A; *F*(1, 41) = 5.33, *p* = 0.026, $\eta_{p}^{2}=0.086$). However, this was not evident in the stress group (*F*(1, 44) = 2.16, *p* = 0.148, $\eta_{p}^{2}=0.03$; subsequent memory × group: *F*(1, 85) = 7.11, *p* = 0.009, $\eta_{p}^{2}=0.054$).

Instead, CA1-EC cofluctuation in the stress group tracked statistical learning. For the purposes of visualization and comparison with episodic encoding, we binarized performance on the pair familiarity test (above-chance accuracy = ‘learned’; below-chance accuracy = ‘not learned’). During A items, CA1-EC cofluctuation in the stress group was significantly higher for learned compared with not learned pairs (figure 4B; *F*(1, 62) = 4.75, *p* = 0.033, $\eta_{p}^{2}=0.048$). This pattern was not evident in the control group (*F*(1, 49) = 0.51, *p* = 0.480, $\eta_{p}^{2}=0.007$; familiarity × group: (*F*(1, 111) = 3.69, *p* = 0.057, $\eta_{p}^{2}=0.022$). A version of this analysis using a continuous measure of pair familiarity strength revealed an even stronger relationship between CA1-EC and statistical learning with stress (electronic supplementary material, figure S6A). Although we did not observe reliable above-chance performance or a difference between groups when pair familiarity test accuracy was averaged across pairs (electronic supplementary material, figure S5), participants in the stress group who performed better on the pair familiarity test had significantly higher CA1-EC connectivity, a pattern that was not evident in the control group (electronic supplementary material, figure S6B).

Consistent with its shared role in both TSP and MSP, these results show that CA1-EC cofluctuation reflects a trade-off between episodic memory and statistical learning. Accordingly, we expect connections exclusive to TSP to support episodic encoding, but not statistical learning. Indeed, neither group showed a positive association between cofluctuation and statistical learning along any TSP connection—namely, EC-DG, EC-CA2/3, DG-CA2/3, and CA2/3-CA1 (electronic supplementary material, figure S7B). In fact, stronger cofluctuation between CA2/3 and CA1 was negatively associated with statistical learning in the control group. By contrast, cofluctuation in CA2/3-CA1, as well as DG-CA2/3, is positively associated with episodic memory in the control group, but not the stress group (electronic supplementary material, figure S7A). Together, these results complement earlier subfield analyses with a circuit-based mechanism by which stress prioritizes processing of statistical regularities at the expense of episodic details (figure 4C).

## 4. Discussion

In this study, we explored how stress, known to impair episodic memory, influences hippocampal processes associated with statistical learning. Across analyses of multivoxel patterns within subfields and connectivity dynamics along hippocampal pathways, we find that acute stress favours hippocampal processes supporting prediction. Specifically, acute stress led to greater representation of predictive category-level features (at the expense of episodic detail) in DG, and strengthened the relationship between CA1-EC connectivity and statistical learning (rather than episodic encoding). These findings complement and extend work in non-human animals on the effects of stress and glucocorticoids in specific hippocampal subfields [22–24,28–34] by showing associations with human learning. We consider each of these mechanisms in turn below.

With respect to pattern separation in DG, representations of predictive scenes in DG became less distinctive and more categorical in the stress group, capturing information that helps predict the upcoming scene. This increased category evidence was associated with stronger hippocampal prediction and weaker episodic memory precision. These results are consistent with past work showing that acute stress shifts memory processing towards gist-like information and away from fine-grained details [60–62], a bias associated with altered DG engram cells in a rodent model [63]. This shift toward generalized memory has been associated with elevated glucocorticoid levels [63,64]; this contrasts with adrenergic activation—another part of the stress response—which is associated with attentional narrowing and memory specificity [65,66]. Although the timing of our stress induction was optimized to coincide with learning-concurrent elevations in cortisol (a timepoint that we have previously shown to be most conducive to statistical learning; [20]), further work is needed to test the roles of different components of the stress response in gist-like representations of predictive information. Notably, our task design is a situation in which a shift toward gist-level processing may be adaptive. Namely, representing predictive stimuli in a gist-like manner (i.e. with categorical features rather than idiosyncratic details) facilitates learning which scene category will occur next. Although a shift toward gist-level encoding is commonly characterized as a transition from hippocampal to cortical systems [60,67], our results reveal that a similar shift can occur across subsystems within the hippocampus [68]. They also suggest that stress may confer benefits in other situations in which identifying links between experiences comes at the expense of remembering individual event details [69].

With respect to CA1-EC coupling, by taking a time-locked approach we showed that the CA1-EC pathway can flexibly support either episodic or statistical learning. We propose that this pathway, common to both MSP (associated with statistical learning) and TSP (associated with episodic encoding), serves as a locus of competition between these two forms of learning [68]. Projections between CA1 and EC are theorized to be less sparse, with lower inhibition and a slower learning rate compared with other projections within TSP, facilitating the extraction of regularities and development of integrated representations across multiple episodes [1]. Thus, impairing TSP may decrease competition between episodic and statistical regularities, allowing for more focused processing of predictive information. Indeed, perturbations in a computational model of the hippocampus show that lesioning connections exclusive to TSP (i.e. not the CA1-EC output pathway which overlaps with MSP) results in a slight enhancement in statistical learning [1]. A similar TSP perturbation also enhances MSP functions in a category learning paradigm, greatly improving categorization performance [70]. Consistent with this framework, we found that stress weakened support for episodic memory along TSP connections while shifting the CA1-EC pathway towards supporting statistical learning in predictive situations. Moreover, weakened pattern separation in DG under stress may lead to more prominent category representation for predictive items spreading through TSP and eventually through CA1-EC. This process may further reinforce category representation in DG, as neural network models show that category representation can spread through EC back to DG through big-loop recurrence [70,71]. These findings underscore the need to consider modulatory factors like stress in neural network models of hippocampal function.

Beyond stress, evaluating hippocampal subfields also allows for precise localization of hippocampal contributions to encoding [68,72]. We found predictive representations of upcoming B categories during A in CA2/3 only, as well as stress-induced category evidence for A only in DG. The lack of prediction or category evidence in CA1 is surprising at first glance, given its role in statistical learning as a key component of MSP. However, prediction is facilitated by pattern completion, enabling retrieval of associated memory representations based on partial information [73,74]. This function has been attributed to CA3, which contains unique recurrent connections forming a highly interconnected autoassociative network [75–77]. Our results contribute to a growing body of experimental evidence in support of this account, including recent findings in 7T fMRI identifying representations of visual predictions only in CA2/3 [57], as well as related evidence of prediction in CA2/3/DG from studies that did not separate these subfields [2,56]. Furthermore, participants in the stress group showed only marginal evidence of category representation in CA1 with a much stronger effect in DG. This deviates from category-learning paradigms, with evidence from both neuroimaging and neural network modelling that CA1 has more similar representations for items within the same category [70,78]. This discrepancy may be related to task demands. For example, CA1 has been theorized to support comparison between predicted and experienced inputs [79], and may support non-directional associative representations [2,73] and prediction error signals [80]. Thus, although CA1 may represent category-level content when initially forming category knowledge, it may instead support predictive comparisons in tasks like the one in the current study involving temporal predictions. We did find evidence that connectivity along MSP supports statistical learning under stress, demonstrating the value of studying MSP-related computations from a more dynamic, circuit-level perspective. Further work is needed to clarify the varied roles of CA1 in learning and representing predictive regularities and how these processes may be modulated by stress.

In addition to successful hippocampal prediction of upcoming scenes, we found that pupil responses during learning reflected sensitivity to predictive regularities. In both groups, pupil size differentiated predictive A and predictable B images. Prediction has well-documented effects on pupil size: unexpected or prediction-violating stimuli in both auditory [81] and visual modalities [82] evoke increased pupil dilation (or less constriction) compared with predicted stimuli, though effects are weaker in the visual modality [83]. Pupil size also plays a role in detecting temporal structure [84] and regularities [85,86]; however, to our knowledge prediction has not previously been measured using pupillary response during statistical learning. As the stimuli in our study were visual, the dominant pupil response is one of constriction due to the change in luminance. Thus, as unpredictable stimuli lead to less constriction, we would expect more constriction for predictable stimuli. We observed this pattern in the control group. However, although the stress group also differentiated predictive and predictable images, the pattern was reversed, with stronger constriction for predictive A items. Given the roles of pupil responses in arousal [87], and interactions between pupil-associated adrenergic responses and other parts of the stress response [88–90], it is reasonable to expect that stress might modulate pupil responses during learning. Previous work suggests that stress exposure can lead to more stimulus-evoked pupil constriction [91] and, in threatening situations, that more constriction reflects more certainty [92]. Furthermore, pupil dilation tracks interactive effects of salience and predictability, with less constriction for predictable elements of salient musical sequences [93]; this may be related to less constriction for predictable B items under stress. Further work is required to disentangle how stress modulates pupil response to learning predictive regularities.

Although our findings suggest that stress enhances hippocampal processing of predictive features, they do not reveal an out-right facilitation of statistical learning. Both the stress and control groups showed distinct pupil responses to A and B images during learning, and neither decoding strength for B categories nor performance on the pair familiarity test showed significant differences between groups. Although the pair familiarity test was insensitive to the stress manipulation at the group level, it had several other advantages. First, by conducting it the day after learning, we isolated stress effects to the extraction of regularities rather than their retrieval [18]. Second, because we wanted to test pair familiarity at the category level, conducting this test offline—after learning had occurred—prevented cueing participants to the category structure such that they would attempt to learn it intentionally. In lieu of behavioural tests during learning, we instead relied on an indirect pupil measure to assess online statistical learning. Third, presenting and assessing regularities at the category level (i.e. showing participants cartoon versions of scene subcategories rather than exact scene images from encoding) made our tasks less prone to overlap between episodic memory and statistical learning behaviours. These tasks also had distinct neural correlates, as pair familiarity performance was associated only with cofluctuation along MSP in the stress group whereas episodic memory performance was associated only with cofluctuation along TSP in the control group.

There were also some drawbacks to the design that may have limited our ability to detect enhancements of statistical learning with stress. In our earlier behavioural study using a similar design, participants exposed to acute stress showed the clearest statistical learning and retention of category-level regularities [20]. One potentially impactful difference between designs concerns the introduction of unstructured information. In order to assess neural evidence of prediction, the current study included an additional unstructured post-learning run after statistical learning but before the pair familiarity test. This unstructured information (i.e. repeated violations of AB pairs) may have caused retroactive interference with learned regularities that were probed the next day [2]. Despite inconsistent behavioural results, the current study builds on our earlier study by revealing a hippocampal mechanism that prioritizes predictive processing after stress. Notably, consistent with the earlier study, participants in the control group demonstrated a behavioural episodic memory enhancement for A items that was not apparent with stress, showing a robust and lasting consequence of statistical regularities for episodic memory in the absence of stress.

Our findings reveal an adaptive role for stress in changing hippocampal mechanisms to prioritize predictive regularities. Un-predictability is a key feature of stressful events [94], with perceived environmental uncertainty modulating acute stress responses [92]. Accordingly, the stress response has been theorized to promote processes that resolve uncertainty [94,95]. Statistical learning may be one such process. It has recently been proposed as a mechanism for acquiring baseline regularities in an environment, enabling rapid detection of deviations that signal environmental change [96]. Therefore, by suppressing encoding of idiosyncratic details and prioritizing neural resources for detecting and representing predictive information, stress may enhance our ability to respond to unexpected events. Finally, our findings add to a growing literature on how the hippocampus supports multiple learning functions across competing hippocampal pathways. We show that these pathways can be differentially modulated by stress, which may uncover opportunities for enhancing learning under stress.

## Supplementary Material

Supplementary figures

Supplementary material is available online at https://doi.org/10.6084/m9.figshare.c.8553591.

## Figures and Tables

**Figure 1.**
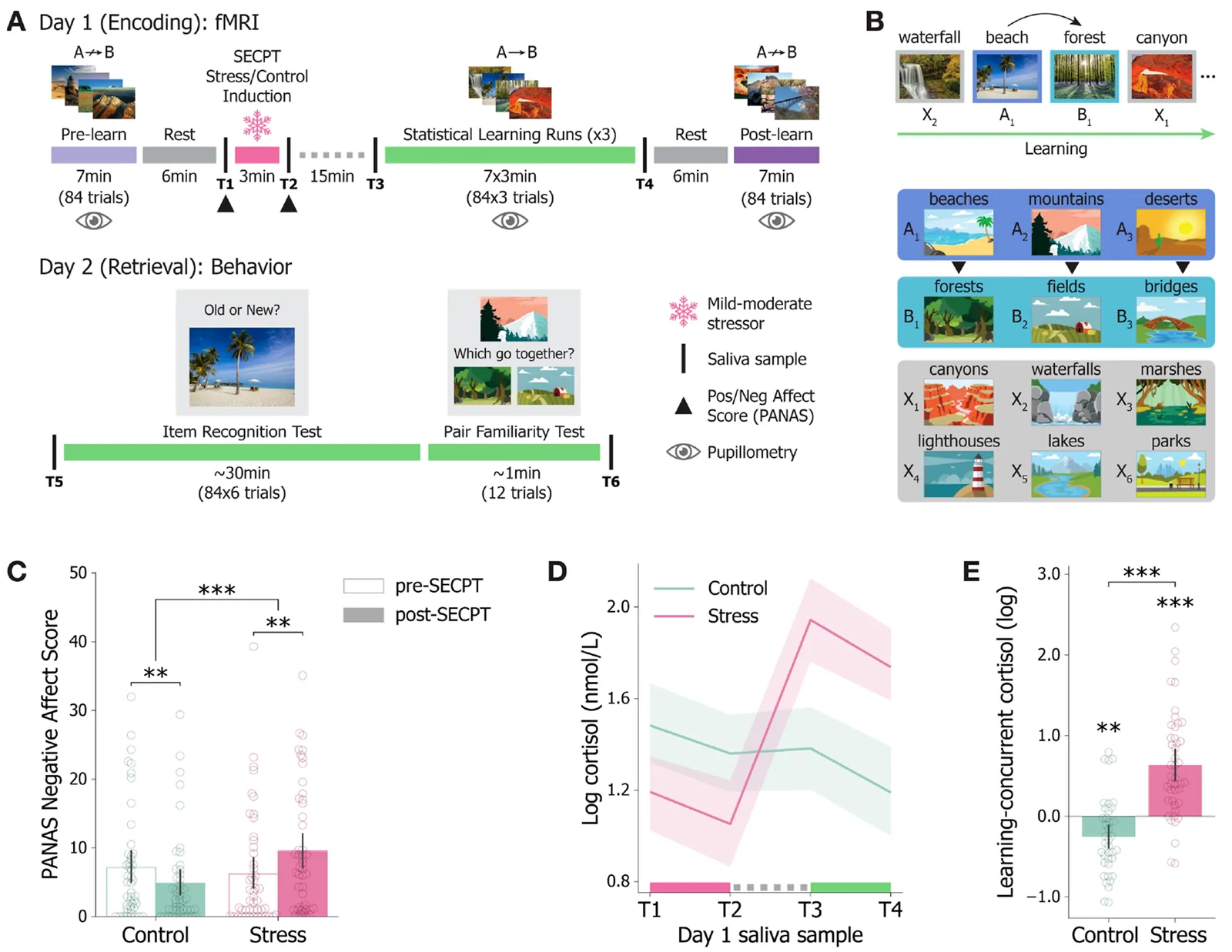
Task design and stress validation. (A) Experiment timeline. (B) Example trial sequence during statistical learning runs (top). A category images were always followed by paired B category images. Example scene categories from A (predictive), B (predictable), and X (baseline) conditions (bottom). (C) Subjective negative affect ratings before and after the socially evaluated cold pressor test (SECPT; pink) or matched control procedure (green). (D) Salivary cortisol throughout day 1: T1 (pre-SECPT); T2 (post-SECPT); T3 (pre-learning); T4 (post-learning). (E) Mean salivary cortisol across learning (T3 and T4) relative to baseline (T1). Dots, individual participants; error bars, bootstrapped 95% CI across participants; ***p < 0.001, **p < 0.01.

**Figure 2.**
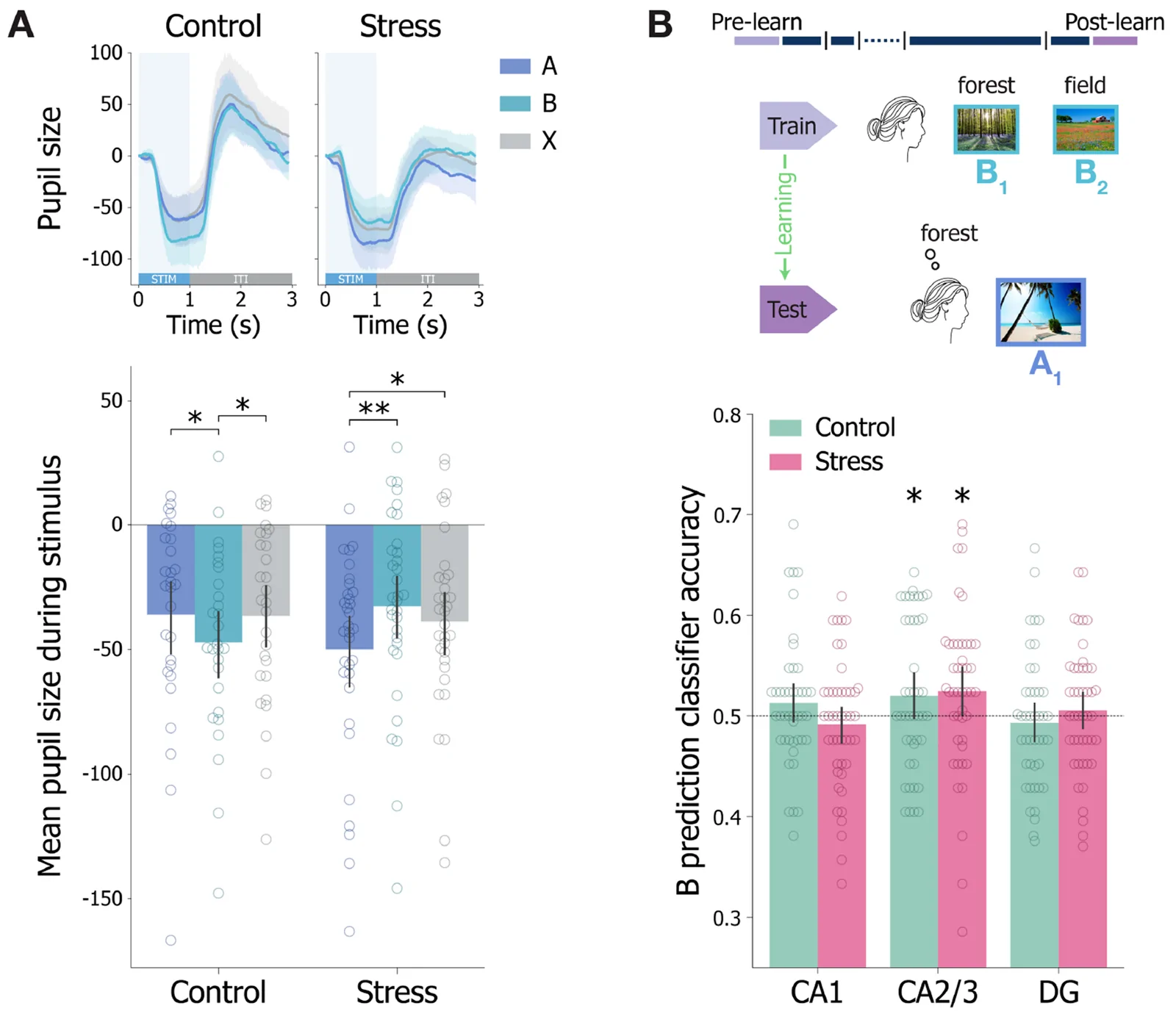
Online and offline evidence of statistical learning. (A) Pupil size for A, B, and X scenes during learning (top: blue shading, stimulus presentation; grey, ITI [intertribal interval]). Mean pupil size during stimulus presentation (bottom). (B) Participant-specific classifiers were trained to decode pairs of B categories using multivoxel patterns from the pre-learning run, then applied to A scenes in the post-learning run to test for prediction of the upcoming B category (top). Classifier accuracy per group for hippocampal subfields CA1, CA2/3 and DG (chance = 0.5) (bottom). Dots, individual participants; error bars, bootstrapped 95% CI across participants; **p < 0.01, *p < 0.05.

**Figure 3.**
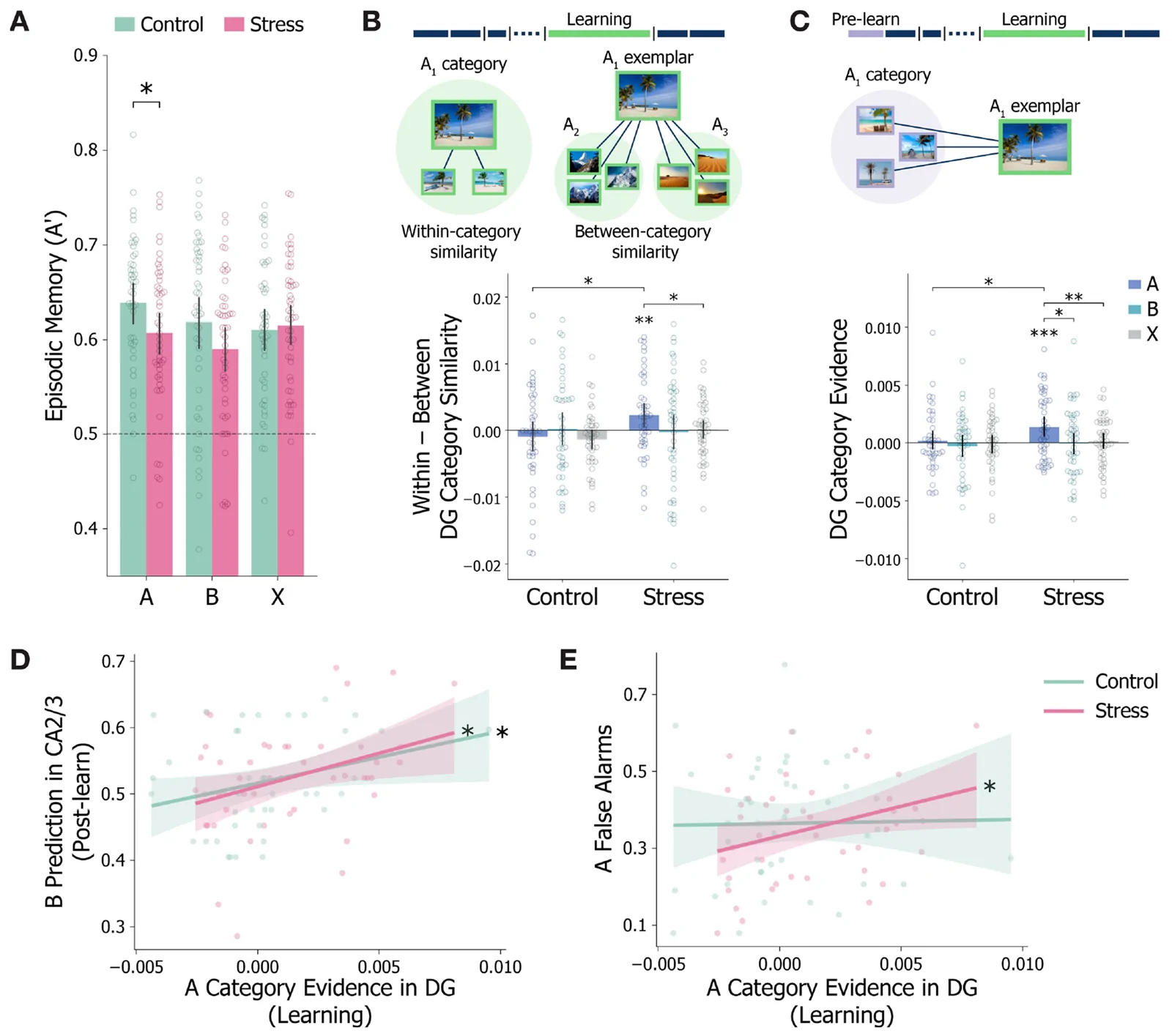
Stress effects on episodic memory. (A) Day 2 item recognition memory for A, B, and X scene conditions. (B) Pattern separation in dentate gyrus (DG) during learning is assessed by calculating similarity of multivoxel patterns for items within the same versus different categories from the same condition (top). Difference scores shown per condition and group; values above zero indicate impaired pattern separation (bottom). (C) Representation of category information in DG is assessed by calculating multivoxel pattern similarity between each (trial-unique) image during learning with images from the same category in the pre-learning run (top). The resulting value is shown per condition and group, with values above zero indicating category evidence (bottom). (D) Relationship between category evidence for A items in DG during learning (see C) and classifier accuracy for the upcoming B item in CA2/3 after learning. (E) Relationship between category evidence for A items in DG during learning and false alarm rate for A items. Dots, individual participants; error bars, bootstrapped 95% CI across participants; ***p < 0.001, **p < 0.01, *p < 0.05.

**Figure 4.**
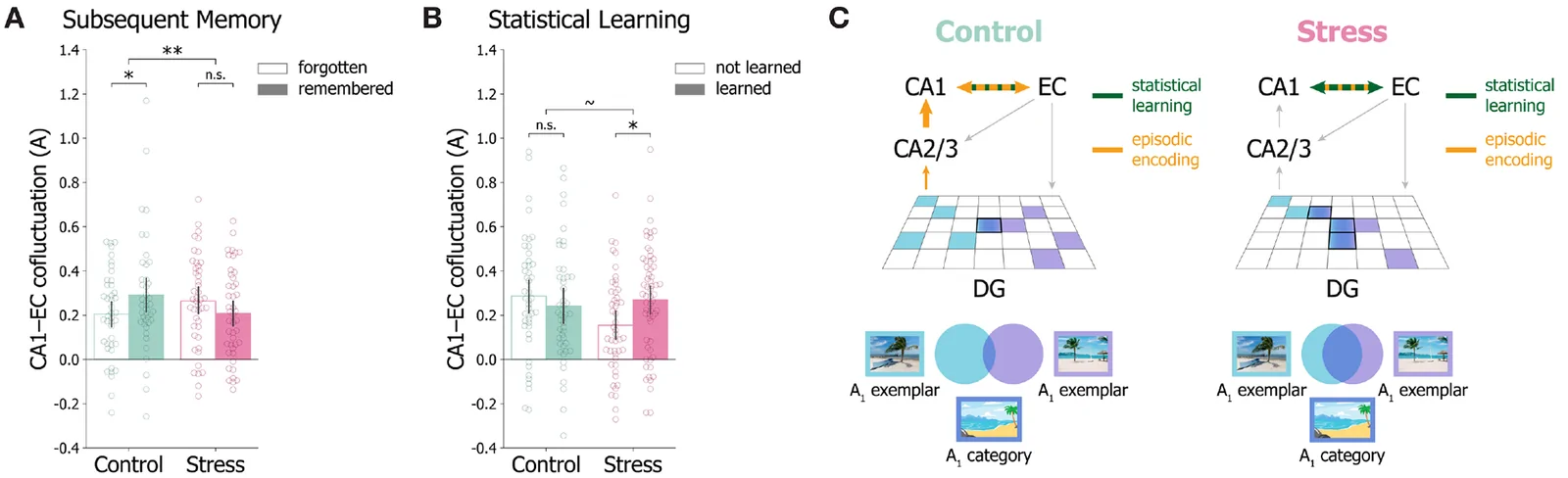
Stress effects on processing in the monosynaptic pathway. (A) Momentary cofluctuation between CA1 and EC for predictive A items that were subsequently remembered and forgotten within each group. Dots, individual participants. (B) Momentary cofluctuation between CA1 and EC for predictive A items belonging to pairs that are either learned (above-chance pair familiarity strength) or not learned (below-chance) within each group. Dots, individual A–B pairs (three per participant). (C) Schematic summarizing the effects of stress on statistical (green) and episodic (yellow) processing of predictive scenes in the hippocampus. In the control group, connectivity between CA1-EC, as well as TSP connections DG-CA2/3 and CA2/3-CA1, relates to episodic memory but not statistical learning (see electronic supplementary material, figure S7). Under stress, connectivity between CA1-EC relates to statistical learning but not episodic memory, and DG representations show increased (predictive) category information. Error bars = 95% CI across participants; **p < 0.01, *p < 0.05, ~*p* < 0.07.

## Data Availability

The data supporting this article are accessible at https://doi.org/10.5281/zenodo.20949110. Code is available at https://doi.org/10.5281/zenodo.20953645 Raw data are also deposited at the NIMH Data Archive Repository (NDAR, Data Collection ID = 4435). Supplementary material is available online [97].

## References

[1] Schapiro AC, Turk-Browne NB, Botvinick MM, Norman KA. 2017 Complementary learning systems within the hippocampus: a neural network modelling approach to reconciling episodic memory with statistical learning. Phil. Trans. R. Soc. B. 372, 20160049. (doi:10.1098/rstb.2016.0049) PMC5124075 27872368

[2] Schapiro AC, Kustner LV, Turk-Browne NB. 2012 Shaping of object representations in the human medial temporal lobe based on temporal regularities. Curr. Biol. 22, 1622–1627. (doi:10.1016/j.cub.2012.06.056) PMC3443305 22885059

[3] Sherman BE, Turk-Browne NB. 2020 Statistical prediction of the future impairs episodic encoding of the present. Proc. Natl Acad. Sci. USA. 117, 22760–22770. (doi:10.1073/pnas.2013291117) PMC7502714 32859755

[4] Bakker A, Kirwan CB, Miller M, Stark CEL. 2008 Pattern separation in the human hippocampal CA3 and dentate gyrus. Science 319, 1640–1642. (doi:10.1126/science.1152882) PMC2829853 18356518

[5] Berron D, Schutze H, Maass A, Cardenas-Blanco A, Kuijf HJ, Kumaran D, Duzel E. 2016 Strong evidence for pattern separation in human dentate gyrus. J. Neurosci. 36, 7569–7579. (doi:10.1523/jneurosci.0518-16.2016) PMC6705559 27445136

[6] Seckl JR, Dickson KL, Yates C, Fink G. 1991 Distribution of glucocorticoid and mineralocorticoid receptor messenger RNA expression in human postmortem hippocampus. Brain Res. 561, 332–337. (doi:10.1016/0006-8993(91)91612-5) 1666329

[7] Lupien SJ, Maheu F, Tu M, Fiocco A, Schramek TE. 2007 The effects of stress and stress hormones on human cognition: implications for the field of brain and cognition. Brain Cogn. 65, 209–237. (doi:10.1016/j.bandc.2007.02.007) 17466428

[8] Wang Q, Van Heerikhuize J, Aronica E, Kawata M, Seress L, Joels M, Swaab DF, Lucassen PJ. 2013 Glucocorticoid receptor protein expression in human hippocampus; stability with age. Neurobiol. Aging 34, 1662–1673. (doi:10.1016/j.neurobiolaging.2012.11.019) 23290588

[9] Cazakoff BN, Howland JG. 2010 Acute stress disrupts paired pulse facilitation and long-term potentiation in rat dorsal hippocampus through activation of glucocorticoid receptors. Hippocampus 20, 1327–1331. (doi:10.1002/hipo.20738) 20043285

[10] Chen F 2020 Structural plasticity and molecular markers in hippocampus of male rats after acute stress. Neuroscience 438, 100–115. (doi:10.1016/j.neuroscience.2020.05.001) 32407976

[11] Chen Y, Dubé CM, Rice CJ, Baram TZ. 2008 Rapid loss of dendritic spines after stress involves derangement of spine dynamics by corticotropin-releasing hormone. J. Neurosci. 28, 2903–2911. (doi:10.1523/jneurosci.0225-08.2008) PMC2409370 18337421

[12] Pruessner JC 2008 Deactivation of the limbic system during acute psychosocial stress: evidence from positron emission tomography and functional magnetic resonance imaging studies. Biol. Psychiatry 63, 234–240. (doi:10.1016/j.biopsych.2007.04.041) 17686466

[13] De Quervain DJ-F. 2003 Glucocorticoid-induced impairment of declarative memory retrieval is associated with reduced blood flow in the medial temporal lobe. Eur. J. Neurosci. 17, 1296–1302. (doi:10.1046/j.1460-9568.2003.02542.x) 12670318

[14] Goldfarb EV, Rosenberg MD, Seo D, Constable RT, Sinha R. 2020 Hippocampal seed connectome-based modeling predicts the feeling of stress. Nat. Commun. 11, 2650. (doi:10.1038/s41467-020-16492-2) PMC7253445 32461583

[15] Quaedflieg CWEM, Schneider TR, Daume J, Engel AK, Schwabe L. 2020 Stress impairs intentional memory control through altered theta oscillations in lateral parietal cortex. J. Neurosci. 7739–7748. (doi:10.1523/jneurosci.2906-19.2020) PMC7531551 32868459

[16] Vaisvaser S 2013 Neural traces of stress: cortisol related sustained enhancement of amygdala–hippocampal functional connectivity. Front. Hum. Neurosci. 7, 313. (doi:10.3389/fnhum.2013.00313) PMC3701866 23847492

[17] Payne JD, Jackson ED, Hoscheidt S, Ryan L, Jacobs WJ, Nadel L. 2007 Stress administered prior to encoding impairs neutral but enhances emotional long-term episodic memories. Learn. Mem. 14, 861–868. (doi:10.1101/lm.743507) PMC2151024 18086830

[18] Shields GS, Sazma MA, McCullough AM, Yonelinas AP. 2017 The effects of acute stress on episodic memory: a meta-analysis and integrative review. Psychol. Bull. 143, 636–675. (doi:10.1037/bul0000100) PMC5436944 28368148

[19] Joëls M, Pu Z, Wiegert O, Oitzl MS, Krugers HJ. 2006 Learning under stress: how does it work? Trends Cogn. Sci. 10, 152–158. (doi:10.1016/j.tics.2006.02.002) 16513410

[20] Sherman BE, Huang I, Wijaya EG, Turk-Browne NB, Goldfarb EV. 2024 Acute stress effects on statistical learning and episodic memory. J. Cogn. Neurosci. 36, 1741–1759. (doi:10.1162/jocn_a_02178) PMC11223726 38713878

[21] Tóth-Fáber E, Janacsek K, Szőllősi Á, Kéri S, Nemeth D. 2021 Regularity detection under stress: Faster extraction of probability-based regularities. PLoS One 16, e0253123. (doi:10.1371/journal.pone.0253123) PMC8205133 34129623

[22] Yap JJ, Takase LF, Kochman LJ, Fornal CA, Miczek KA, Jacobs BL. 2006 Repeated brief social defeat episodes in mice: effects on cell proliferation in the dentate gyrus. Behav. Brain Res. 172, 344–350. (doi:10.1016/j.bbr.2006.05.027) 16839619

[23] Gould E, McEwen BS, Tanapat P, Galea LAM, Fuchs E. 1997 Neurogenesis in the dentate gyrus of the adult tree shrew is regulated by psychosocial stress and NMDA receptor activation. J. Neurosci. 17, 2492–2498. (doi:10.1523/jneurosci.17-07-02492.1997) PMC6573503 9065509

[24] Thomas RM, Hotsenpiller G, Peterson DA. 2007 Acute psychosocial stress reduces cell survival in adult hippocampal neurogenesis without altering proliferation. J. Neurosci. 27, 2734–2743. (doi:10.1523/jneurosci.3849-06.2007) PMC6672591 17360895

[25] Tuncdemir SN, Lacefield CO, Hen R. 2019 Contributions of adult neurogenesis to dentate gyrus network activity and computations. Behav. Brain Res. 374, 112112. (doi:10.1016/j.bbr.2019.112112) PMC6724741 31377252

[26] Sahay A, Scobie KN, Hill AS, O’Carroll CM, Kheirbek MA, Burghardt NS, Fenton AA, Dranovsky A, Hen R. 2011 Increasing adult hippocampal neurogenesis is sufficient to improve pattern separation. Nature 472, 466–470. (doi:10.1038/nature09817) PMC3084370 21460835

[27] Clelland CD 2009 A functional role for adult hippocampal neurogenesis in spatial pattern separation. Science 325, 210–213. (doi:10.1126/science.1173215) PMC2997634 19590004

[28] Pavlides C, Watanabe Y, McEwen BS. 1993 Effects of glucocorticoids on hippocampal long-term potentiation. Hippocampus 3, 183–192. (doi:10.1002/hipo.450030210) 8353605

[29] Yamada K, McEwen BS, Pavlides C. 2003 Site and time dependent effects of acute stress on hippocampal long-term potentiation in freely behaving rats. Exp. Brain Res. 152, 52–59. (doi:10.1007/s00221-003-1519-0) 12879172

[30] Chen CC, Yang CH, Huang CC, Hsu KS. 2010 Acute stress impairs hippocampal mossy fiber-CA3 long-term potentiation by enhancing cAMP-specific phosphodiesterase 4 activity. Neuropsychopharmacology 35, 1605–1617. (doi:10.1038/npp.2010.33) PMC3055459 20237461

[31] Wiegert O, Pu Z, Shor S, Joëls M, Krugers H. 2005 Glucocorticoid receptor activation selectively hampers N-methyl-D-aspartate receptor dependent hippocampal synaptic plasticity in vitro. Neuroscience 135, 403–411. (doi:10.1016/j.neuroscience.2005.05.039) 16125856

[32] Karst H, Joëls M. 2005 Corticosterone slowly enhances miniature excitatory postsynaptic current amplitude in mice CA1 hippocampal cells. J. Neurophysiol. 94, 3479–3486. (doi:10.1152/jn.00143.2005) 16033944

[33] Vandael D, Wierda K, Vints K, Baatsen P, De Groef L, Moons L, Rybakin V, Gounko NV. 2021 Corticotropin-releasing factor induces functional and structural synaptic remodelling in acute stress. Transl. Psychiatry 11, 378. (doi:10.1038/s41398-021-01497-2) PMC8263770 34234103

[34] van Gemert NG, Carvalho DMM, Karst H, van der Laan S, Zhang M, Meijer OC, Hell JW, Joëls M. 2009 Dissociation between rat hippocampal CA1 and dentate gyrus cells in their response to corticosterone: effects on calcium channel protein and current. Endocrinology 150, 4615–4624. (doi:10.1210/en.2009-0525) PMC2754681 19589863

[35] Goldfarb EV. 2019 Enhancing memory with stress: progress, challenges, and opportunities. Brain Cogn. 133, 94–105. (doi:10.1016/j.bandc.2018.11.009) PMC9972486 30553573

[36] Cohen S, Kamarck T, Mermelstein R. 1983 A global measure of perceived stress. J. Health Soc. Behav. 24, 385–396. (doi:10.2307/2136404) 6668417

[37] Linden W, McEachern HM. 1985 A review of physiological prestress adaptation: effects of duration and context. Int. J. Psychophysiol. 2, 239–245. (doi:10.1016/0167-8760(85)90002-9) 3888937

[38] Schwabe L, Haddad L, Schachinger H. 2008 HPA axis activation bya socially evaluated cold-pressor test. Psychoneuroendocrinology 33, 890–895. (doi:10.1016/j.psyneuen.2008.03.001) 18403130

[39] Schwabe L, Schächinger H. 2018 Ten years of research with the socially evaluated cold pressor test: data from the past and guidelines for the future. Psychoneuroendocrinology 92, 155–161. (doi:10.1016/j.psyneuen.2018.03.010) 29573884

[40] Dressendörfer RA, Kirschbaum C, Rohde W, Stahl F, Strasburger CJ. 1992 Synthesis of a cortisol-biotin conjugate and evaluation as a tracer in an immunoassay for salivary cortisol measurement. J. Steroid Biochem. Mol. Biol. 43, 683–692. (doi:10.1016/0960-0760(92)90294-s) 1472460

[41] Goldfarb EV, Froböse MI, Cools R, Phelps EA. 2017 Stress and cognitive flexibility: cortisol increases are associated with enhanced updating but impaired switching. J. Cogn. Neurosci. 29,14–24. (doi:10.1162/jocn_a_01029) 27576026

[42] Pinheiro J, Bates D, R Core Team. 1999 nlme: linear and nonlinear mixed effects models. R package version 3.1–168. (doi:10.32614/cran.package.nlme)

[43] Lüdecke D. 2025 sjstats: statistical functions for regression models (Version 0.19.1). Zenodo. (doi:10.5281/zenodo.1284472)

[44] Grier JB. 1971 Nonparametric indexes for sensitivity and bias: computing formulas. Psychol. Bull. 75, 424–429. (doi:10.1037/h0031246) 5580548

[45] Geller J, Winn MB, Mahr T, Mirman D. 2020 GazeR: a package for processing gaze position and pupil size data. Behav. Res. Methods. 52, 2232–2255. (doi:10.3758/s13428-020-01374-8) PMC7544668 32291732

[46] Mathôt S, Fabius J, Van Heusden E, Van der Stigchel S. 2018 Safe and sensible preprocessing and baseline correction of pupil-size data. Behav. Res. Methods 50, 94–106. (doi:10.3758/s13428-017-1007-2) PMC5809553 29330763

[47] Jenkinson M, Bannister P, Brady M, Smith S. 2002 Improved optimization for the robust and accurate linear registration and motion correction of brain images. Neuroimage 17, 825–841. (doi:10.1006/nimg.2002.1132) 12377157

[48] Yushkevich PA 2015 Automated volumetry and regional thickness analysis of hippocampal subfields and medial temporal cortical structures in mild cognitive impairment. Hum. Brain Mapp. 36, 258–287. (doi:10.1002/hbm.22627) PMC4313574 25181316

[49] Aly M, Turk-Browne NB. 2016 Attention stabilizes representations in the human hippocampus. Cereb. Cortex 26, 783–796. (doi:10.1093/cercor/bhv041) PMC4712804 25766839

[50] Zamani Esfahlani F, Jo Y, Faskowitz J, Byrge L, Kennedy DP, Sporns O, Betzel RF. 2020 High-amplitude cofluctuations in cortical activity drive functional connectivity. Proc. Natl Acad Sci. USA 117, 28393–28401. (doi:10.1073/pnas.2005531117) PMC7668041 33093200

[51] Faskowitz J, Esfahlani FZ, Jo Y, Sporns O, Betzel RF. 2020 Edge-centric functional network representations of human cerebral cortex reveal overlapping system-level architecture. Nat. Neurosci. 23, 1644–1654. (doi:10.1038/s41593-020-00719-y) 33077948

[52] Al-Aidroos N, Said CP, Turk-Browne NB. 2012 Top-down attention switches coupling between low-level and high-level areas of human visual cortex. Proc. Natl Acad. Sci. USA 109, 14675–14680. (doi:10.1073/pnas.1202095109) PMC3437858 22908274

[53] Norman-Haignere SV, McCarthy G, Chun MM, Turk-Browne NB. 2012 Category-selective background connectivity in ventral visual cortex. Cereb. Cortex 22, 391–402. (doi:10.1093/cercor/bhr118) PMC3256407 21670097

[54] Córdova NI, Tompary A, Turk-Browne NB. 2016 Attentional modulation of background connectivity between ventral visual cortex and the medial temporal lobe. Neurobiol. Learn. Mem.134,115–122. (doi:10.1016/j.nlm.2016.06.011) PMC5018439 27321163

[55] Efron B, Tibshirani R. 1986 Bootstrap methods for standard errors, confidence intervals, and other measures of statistical accuracy. Stat. Sci. 1, 54–75. (doi:10.1214/ss/1177013815)

[56] Kok P, Turk-Browne NB. 2018 Associative prediction of visual shape in the hippocampus. J. Neurosci. 38, 6888–6899. (doi:10.1523/jneurosci.0163-18.2018) PMC6070666 29986875

[57] Warrington O, Graedel NN, Callaghan MF, Kok P. 2025 Communication of perceptual predictions from the hippocampus to the deep layers of the parahippocampal cortex. Sci. Adv. 11, eads4970. (doi:10.1126/sciadv.ads4970) PMC12094225 40397746

[58] McClelland JL, McNaughton BL, O’Reilly RC. 1995 Why there are complementary learning systems in the hippocampus and neocortex: insights from the successes and failures of connectionist models of learning and memory. Psychol. Rev. 102, 419–457. (doi:10.1037/0033-295x.102.3.419) 7624455

[59] Brunec IK, Robin J, Olsen RK, Moscovitch M, Barense MD. 2020 Integration and differentiation of hippocampal memory traces. Neurosci. Biobehav. Rev. 118, 196–208. (doi:10.1016/j.neubiorev.2020.07.024) 32712280

[60] Qin S, Hermans EJ, van Marle HJF, Fernández G. 2012 Understanding low reliability of memories for neutral information encoded under stress: alterations in memory-related activation in the hippocampus and midbrain. J. Neurosci. 32, 4032–4041. (doi:10.1523/jneurosci.3101-11.2012) PMC6621211 22442069

[61] Payne JD, Nadel L, Allen JJB, Thomas KGF, Jacobs WJ. 2002 The effects of experimentally induced stress on false recognition. Memory 10, 1–6. (doi:10.1080/09658210143000119) 11747571

[62] Christianson S, Loftus EF. 1987 Memory for traumatic events. Appl. Cogn. Psychol. 1, 225–239. (doi:10.1002/acp.2350010402)

[63] Lesuis SL 2021 Glucocorticoids promote fear generalization by increasing the size of a dentate gyrus engram cell population. Biol. Psychiatry 90, 494–504. (doi:10.1016/j.biopsych.2021.04.010) 34503674

[64] Lesuis SL 2025 Stress disrupts engram ensembles in lateral amygdala to generalize threat memory in mice. Cell 188, 121–140. (doi:10.1016/j.cell.2024.10.034) PMC11726195 39549697

[65] Roozendaal B, Mirone G. 2020 Opposite effects of noradrenergic and glucocorticoid activation on accuracy of an episodic-like memory. Psychoneuroendocrinology 114, 104588. (doi: 10.1016/j.psyneuen.2020.104588) 32085987

[66] Clewett D, Murty VP. 2019 Echoes of emotions past: how neuromodulators determine what we recollect. eNeuro 6, ENEURO.0108-18.2019. (doi:10.1523/ENEURO.0108-18.2019) PMC6437660 30923742

[67] Vogel S, Kluen LM, Fernández G, Schwabe L. 2018 Stress leads to aberrant hippocampal involvement when processing schema-related information. Learn. Mem. 25, 21–30. (doi:10.1101/lm.046003.117) PMC5733469 29246978

[68] Sherman BE, Turk-Browne NB, Goldfarb EV. 2024 Multiple memory subsystems: reconsidering memory in the mind and brain. Perspect. Psychol. Sci. 19, 103–125. (doi:10.1177/17456916231179146) PMC10756937 37390333

[69] Carpenter AC, Schacter DL. 2017 Flexible retrieval: when true inferences produce false memories. J. Exp. Psychol. Learn. Mem. Cogn. 43, 335–349. (doi:10.1037/xlm0000340) PMC5334182 27918169

[70] Sučević J, Schapiro AC. 2023 A neural network model of hippocampal contributions to category learning. eLife 12, e77185. (doi:10.7554/eLife.77185) PMC10712951 38079351

[71] Koster R, Chadwick MJ, Chen Y, Berron D, Banino A, Düzel E, Hassabis D, Kumaran D. 2018 Big-loop recurrence within the hippocampal system supports integration of information across episodes. Neuron 99, 1342–1354. (doi:10.1016/j.neuron.2018.08.009) 30236285

[72] Duncan KD, Schlichting ML. 2018 Hippocampal representations as a function of time, subregion, and brain state. Neurobiol. Learn. Mem. 153, 40–56. (doi:10.1016/j.nlm.2018.03.006) 29535044

[73] Hindy NC, Ng FY, Turk-Browne NB. 2016 Linking pattern completion in the hippocampus to predictive coding in visual cortex. Nat. Neurosci. 19, 665–667. (doi:10.1038/nn.4284) PMC4948994 27065363

[74] Barron HC, Auksztulewicz R, Friston K. 2020 Prediction and memory: a predictive coding account. Prog. Neurobiol. 192, 101821. (doi:10.1016/j.pneurobio.2020.101821) PMC7305946 32446883

[75] Grande X, Berron D, Horner AJ, Bisby JA, Düzel E, Burgess N. 2019 Holistic recollection via pattern completion involves hippocampal subfield CA3. J. Neurosci. 39, 8100–8111. (doi:10.1523/jneurosci.0722-19.2019) PMC6786823 31405925

[76] Rolls ET. 2013 The mechanisms for pattern completion and pattern separation in the hippocampus. Front. Syst. Neurosci. 7, 74. (doi:10.3389/fnsys.2013.00074) PMC3812781 24198767

[77] Treves A, Rolls ET. 1994 Computational analysis of the role of the hippocampus in memory. Hippocampus 4, 374–391. (doi:10.1002/hipo.450040319) 7842058

[78] Schapiro AC, McDevitt EA, Rogers TT, Mednick SC, Norman KA. 2018 Human hippocampal replay during rest prioritizes weakly learned information and predicts memory performance. Nat. Commun. 9, 3920. (doi:10.1038/s41467-018-06213-1) PMC6156217 30254219

[79] Chen J, Olsen RK, Preston AR, Glover GH, Wagner AD. 2011 Associative retrieval processes in the human medial temporal lobe: hippocampal retrieval success and CA1 mismatch detection. Learn. Mem. 18, 523–528. (doi:10.1101/lm.2135211) PMC3256570 21775513

[80] Duncan K, Ketz N, Inati SJ, Davachi L. 2012 Evidence for area CA1 as a match/mismatch detector: a high-resolution fMRI study of the human hippocampus. Hippocampus 22, 389–398. (doi:10.1002/hipo.20933) PMC3529001 21484934

[81] Milne AE, Zhao S, Tampakaki C, Bury G, Chait M. 2021 Sustained pupil responses are modulated by predictability of auditory sequences. J. Neurosci. 41, 6116–6127. (doi:10.1523/jneurosci.2879-20.2021) PMC8276747 34083259

[82] Zhang F, Jaffe-Dax S, Wilson RC, Emberson LL. 2019 Prediction in infants and adults: a pupillometry study. Dev. Sci. 22, e12780. (doi:10.1111/desc.12780) PMC7294578 30506618

[83] Klingner J, Tversky B, Hanrahan P. 2011 Effects of visual and verbal presentation on cognitive load in vigilance, memory, and arithmetic tasks. Psychophysiology 48, 323–332. (doi:10.1111/j.1469-8986.2010.01069.x) 20718934

[84] Clewett D, Gasser C, Davachi L. 2020 Pupil-linked arousal signals track the temporal organization of events in memory. Nat. Commun. 11, 4007. (doi:10.1038/s41467-020-17851-9) PMC7421896 32782282

[85] Akdoğan B, Balcı F, van Rijn H. 2016 Temporal expectation indexed by pupillary response. Timing Time Percept. 4, 354–370. (doi:10.1163/22134468-00002075)

[86] Schwiedrzik CM, Sudmann SS. 2020 Pupil diameter tracks statistical structure in the environment to increase visual sensitivity. J. Neurosci. 40, 4565–4575. (doi:10.1523/jneurosci.0216-20.2020) PMC7275858 32371603

[87] Bradley MM, Miccoli L, Escrig MA, Lang PJ. 2008 The pupil as a measure of emotional arousal and autonomic activation. Psychophysiology 45, 602–607. (doi:10.1111/j.1469-8986.2008.00654.x) PMC3612940 18282202

[88] Reimer J, McGinley MJ, Liu Y, Rodenkirch C, Wang Q, McCormick DA, Tolias AS. 2016 Pupil fluctuations track rapid changes in adrenergic and cholinergic activity in cortex. Nat. Commun. 7,13289. (doi:10.1038/ncomms13289) PMC5105162 27824036

[89] Alexander JK, Hillier A, Smith RM, Tivarus ME, Beversdorf DQ. 2007 Beta-adrenergic modulation of cognitive flexibility during stress. J. Cogn. Neurosci. 19, 468–478. (doi:10.1162/jocn.2007.19.3.468) 17335395

[90] Birnbaum S, Gobeske KT, Auerbach J, Taylor JR, Arnsten AFT. 1999 A role for norepinephrine in stress-induced cognitive deficits: α-1-adrenoceptor mediation in the prefrontal cortex. Biol. Psychiatry 46, 1266–1274. (doi:10.1016/s0006-3223(99)00138-9) 10560032

[91] Henckens M, Hermans EJ, Pu Z, Joëls M, Fernández G. 2009 Stressed memories: how acute stress affects memory formation in humans. J. Neurosci. 29, 10111–10119. (doi:10.1523/JNEUROSCI.1184-09.2009) PMC6664979 19675245

[92] de Berker AO, Rutledge RB, Mathys C, Marshall L, Cross GF, Dolan RJ, Bestmann S. 2016 Computations of uncertainty mediate acute stress responses in humans. Nat. Commun. 7, 10996. (doi:10.1038/ncomms10996) PMC4820542 27020312

[93] Bianco R, Gold BP, Johnson AP, Penhune VB. 2019 Music predictability and liking enhance pupil dilation and promote motor learning in non-musicians. Sci. Rep. 9, 17060. (doi:10.1038/s41598-019-53510-w) PMC6863863 31745159

[94] Peters A, McEwen BS, Friston K. 2017 Uncertainty and stress: why it causes diseases and how it is mastered by the brain. Prog. Neurobiol. 156, 164–188. (doi:10.1016/j.pneurobio.2017.05.004) 28576664

[95] Trapp S, O’Doherty JP, Schwabe L. 2018 Stressful events as teaching signals for the brain. Trends Cogn. Sci. 22, 475–478. (doi:10.1016/j.tics.2018.03.007) 29655607

[96] Frost R, Bogaerts L, Samuel AG, Magnuson JS, Holt LL, Christiansen MH. 2025 Statistical learning subserves a higher purpose: novelty detection in an information foraging system. Psychol. Rev. 133, 237–252. (doi:10.1037/rev0000547) 39992791

[97] ZhouI 2026 Supplementary material from: Stress drives the hippocampus to prioritize statistical prediction over episodic encoding. Figshare. (doi:10.6084/m9.figshare.c.8553591)42421579

