# Supplementary material for: Stress drives the hippocampus to prioritize statistical prediction over episodic encoding
Source: Philos Trans R Soc Lond B Biol Sci. Author manuscript; Available in PMC 2026 Oct 16. (PMC13673394; doi:10.1098/rstb.2025.0238)
Supplement: Supplementary file 1 [file NIHMS2213619-supplement-Supplementary_figures.pdf]

Supplementary material for:
Stress drives the hippocampus to prioritize statistical prediction over episodic encoding

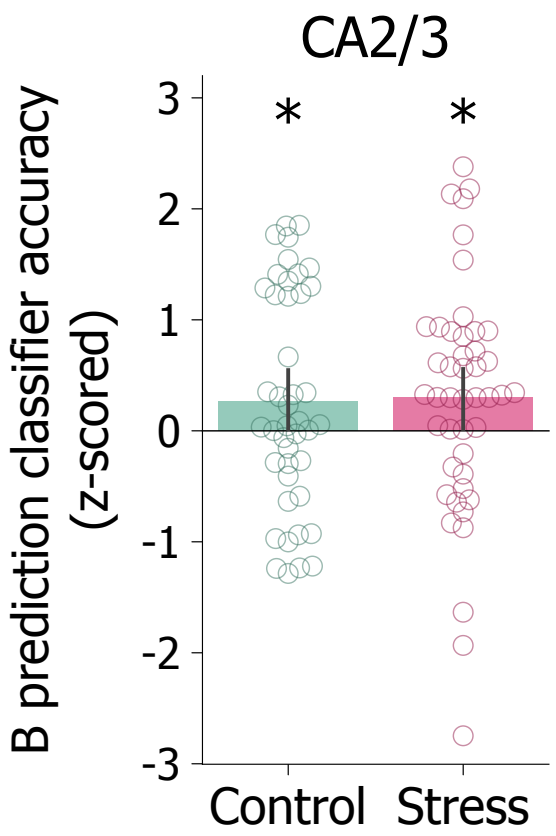

Figure S1. Predictive category decoding analysis with randomization testing. Z-scored classifier accuracies for hippocampal subfield CA2/3 are displayed for each group (see also main text Fig. 2B). Classifier accuracies are z-scored relative to participant-specific null distributions of accuracy values. Z-scored classifier accuracies are reliably above zero for participants in both control (95% CI: $[-0.018, 0.559], p = 0.033, r = 0.313$) and stress (95% CI: $[-0.005, 0.597], p = 0.027, r = 0.382$) groups. Dots = z-scored classification accuracies from individual participants. Error bars = bootstrapped 95% CIs across participants. $^{*}p < 0.05$

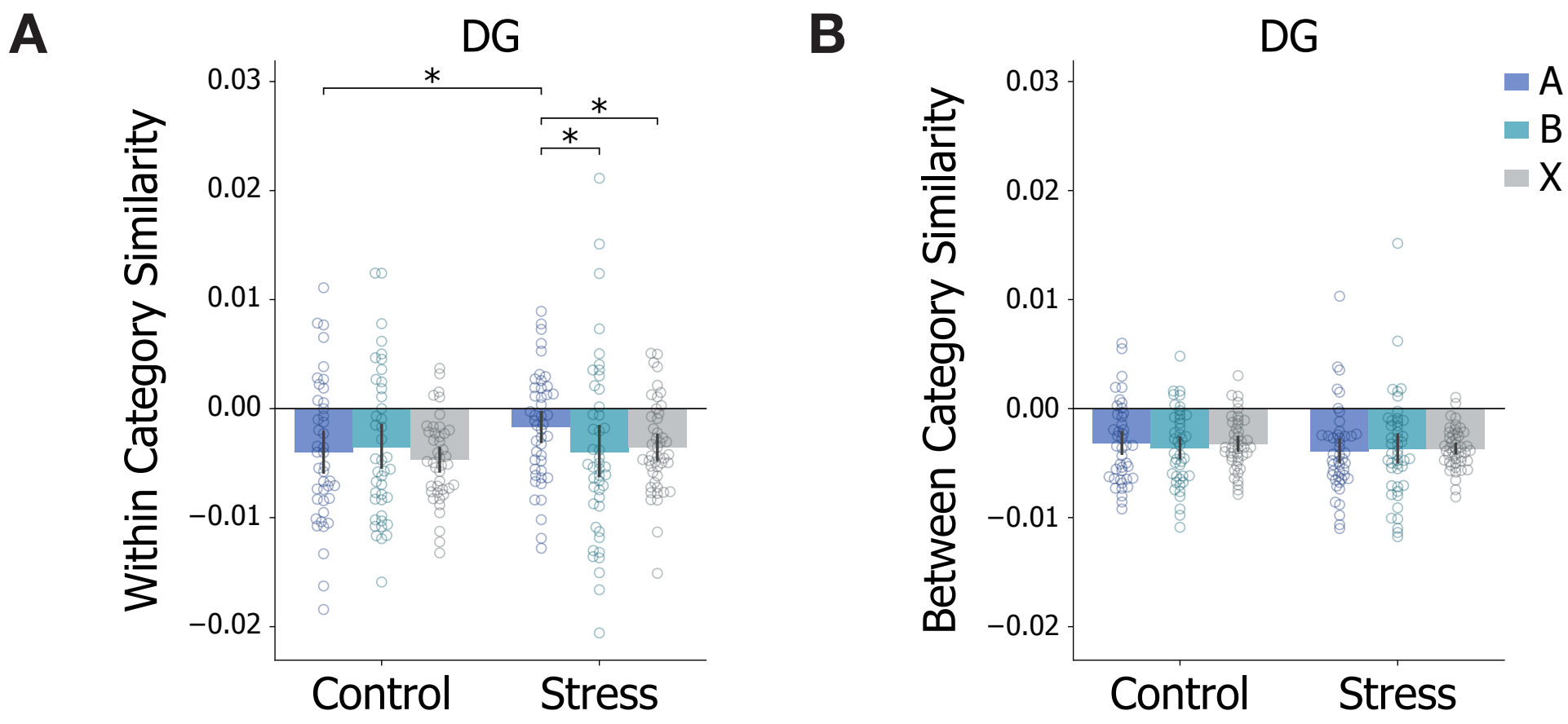

Figure S2. Separate within-category and between-category similarity in DG. The difference between these measures is shown in main text Fig. 3B. Representation similarity is shown for each condition and group. **(A)** Within-category similarity results are similar to those in Fig. 3B. Within the stress group, scenes from the A condition show significantly greater within-category similarity compared to scenes from the B (95% CI: $[-0.31, 4.92] \times 10^{-3}, p = 0.042, r = 0.293$) or X (95% CI: $[0.22, 2.58] \times 10^{-3}, p = 0.014, r = 0.337$) conditions. Within-category similarity for A is also significantly stronger with stress compared to the control group (95% CI: $[0.10, 4.77] \times 10^{-3}, p = 0.031, r = 0.247$). **(B)** Between-category similarity reveals no significant differences across conditions and groups. Dots = individual participants. Error bars = bootstrapped 95% CIs. $^*p < 0.05$

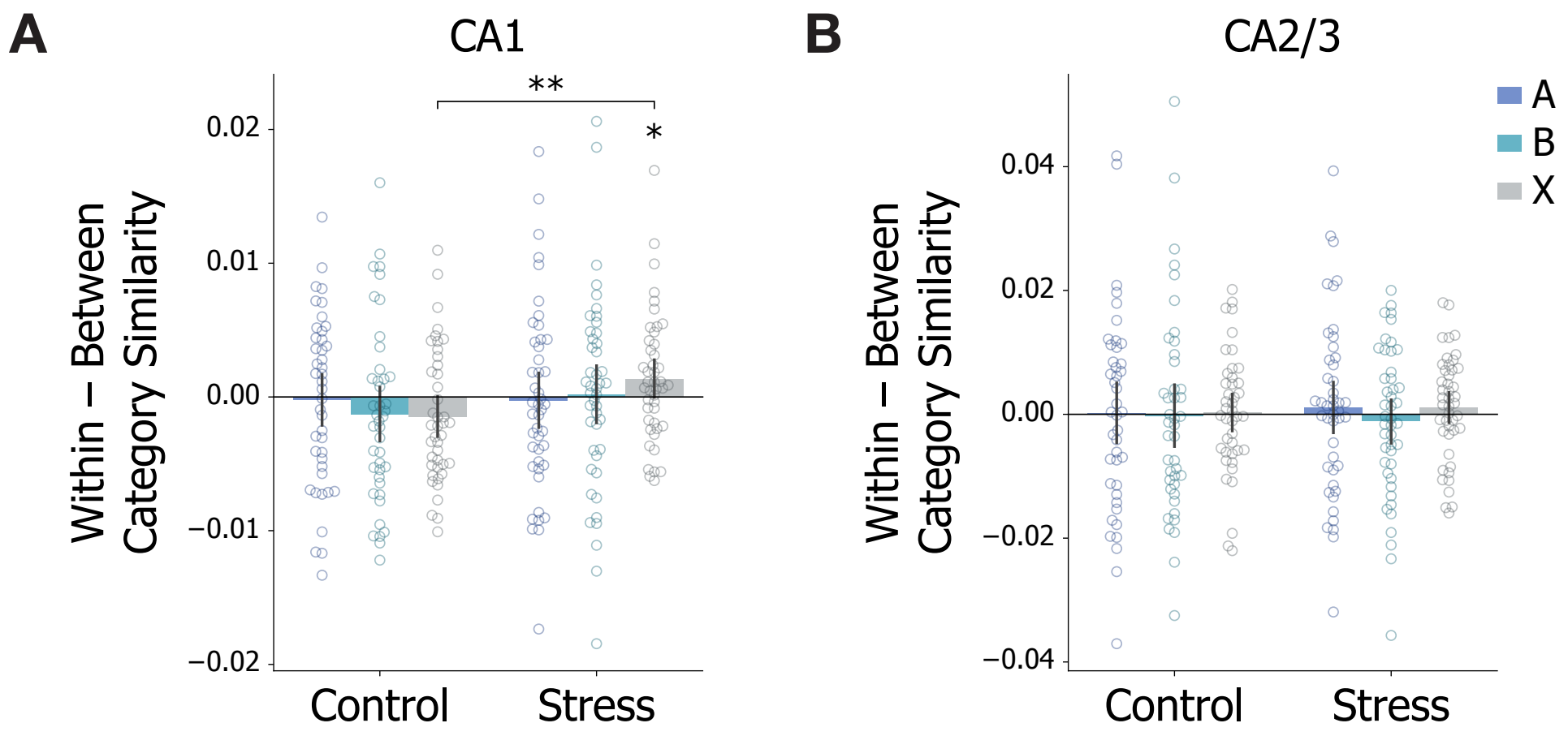

Figure S3. Category representation similarity analysis applied to hippocampal subfields CA1 and CA2/3. DG results shown in main text Fig. 3B. The difference between within- and between-group pattern similarity during learning is shown for each condition and group. **(A)** In CA1, this difference is not significantly greater than zero for any condition in the control group (A: 95% CI: $[-2.15, 1.67] \times 10^{-3}, p = 0.591, r = -0.023$; B: 95% CI: $[-3.29, 0.69] \times 10^{-3}, p = 0.903, r = -0.276$; X: 95% CI: $[-2.97, 0.03] \times 10^{-3}, p = 0.972, r = -0.351$), and for the A and B conditions in the stress group (A: 95% CI: $[-2.26, 1.82] \times 10^{-3}, p = 0.597, r = -0.074$; B: 95$ CI: $[-1.94, 2.33] \times 10^{-3}, p = 0.437, r = 0.038$). However, the difference is significantly higher for the X condition in the stress group (95% CI: $[0.00, 2.77] \times 10^{-3}, p = 0.025$, corrected $p = 0.049$, $r = 0.264$), compared with the X condition in the control group (95% CI: $[0.81, 4.89] \times 10^{-3}, p = 0.003$, corrected $p = 0.006$, $r = 0.325$). **(B)** In CA2/3, this difference is not significantly greater than zero for any condition in the control (A: 95% CI: $[-4.62, 5.05] \times 10^{-3}, p = 0.476, r = -0.008$; B: 95% CI: $[-5.07, 4.77] \times 10^{-3}, p = 0.557, r = -0.121$; X: 95% CI: $[-2.65, 3.20] \times 10^{-3}, p = 0.424, r = 0.025$) or stress (A: 95% CI: $[-2.92, 5.31] \times 10^{-3}, p = 0.293, r = 0.098$; B: 95$ CI: $[-4.68, 2.28] \times 10^{-3}, p = 0.733, r = -0.045$; X: 95% CI: $[-1.32, 3.56] \times 10^{-3}, p = 0.182, r = 0.175$) groups. Dots = individual participants. Error bars = bootstrapped 95% CIs. Additional Holm-Bonferroni corrected p-values reported for significant or marginal effects. **$p < 0.01$, *$p < 0.05$ (uncorrected)

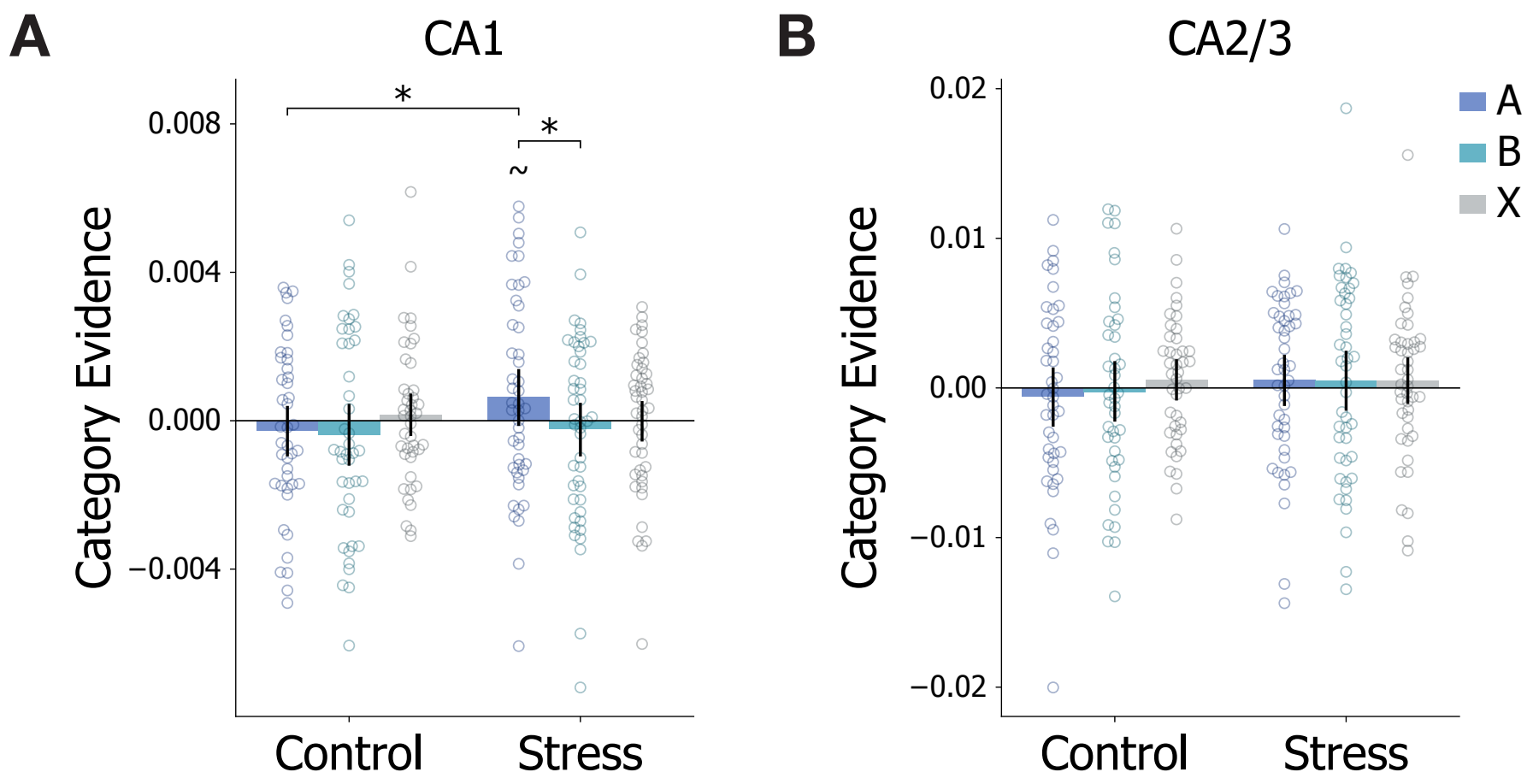

Figure S4. Category evidence analysis applied to hippocampal subfields CA1 and CA2/3, with category evidence shown for each condition and group. **(A)** Category evidence in CA1 was not significantly greater than zero in any condition in the control group (A: 95% CI: $[-0.97, 0.41] \times 10^{-3}, p = 0.778, r = -0.114$; B: 95$ CI: $[-1.20, 0.46] \times 10^{-3}, p = 0.815, r = -0.143$; X: 95% CI: $[-0.41, 0.74] \times 10^{-3}, p = 0.317, r = 0.003$). The stress group showed marginally significant CA1 category evidence for predictive A scenes (95% CI: $[-0.14, 1.42] \times 10^{-3}, p = 0.054$, corrected $p = 0.108$, $r = 0.223$), but not for B (95% CI: $[-0.96, 0.48] \times 10^{-3}, p = 0.730, r = -0.088$) or X (95% CI: $[-0.57, 0.54] \times 10^{-3}, p = 0.494, r = 0.065$). CA1 category evidence for A was significantly stronger than for B (95% CI: $[-0.10, 1.87] \times 10^{-3}, p = 0.041$, corrected $p = 0.081$, $r = 0.219$) but not X (95% CI: $[-0.34, 1.63] \times 10^{-3}, p = 0.101$, corrected $p = 0.202$, $r = 0.206$) within the stress group, and was also significantly stronger than category evidence for A in the control group (95% CI: $[-0.13, 1.95] \times 10^{-3}, p = 0.043$, corrected $p = 0.087$, $r = 0.179$). This pattern of results is similar to (but weaker than) those found in DG (see main text Fig. 3C), and suggest that CA1 may also partially represent the category of predictive items under stress. **(B)** Category evidence in CA2/3 was not significantly greater than zero in any condition for either the control (A: 95% CI: $[-2.48, 1.32] \times 10^{-3}, p = 0.715, r = -0.070$; B: 95% CI: $[-2.20, 1.67] \times 10^{-3}, p = 0.611, r = -0.056$; X: 95% CI: $[-0.74, 1.81] \times 10^{-3}, p = 0.206, r = 0.116$) or stress (A: 95% CI: $[-1.08, 2.12] \times 10^{-3}, p = 0.243, r = 0.181$; B: 95% CI: $[-1.47, 2.38] \times 10^{-3}, p = 0.322, r = 0.084$; X: 95% CI: $[-0.99, 1.92] \times 10^{-3}, p = 0.265, r = 0.129$) groups. Dots = individual participants. Error bars = bootstrapped 95% CIs. Additional Holm-Bonferroni corrected p-values reported for significant or marginal effects. *$p < 0.05$, ~$p < 0.07$ (uncorrected)

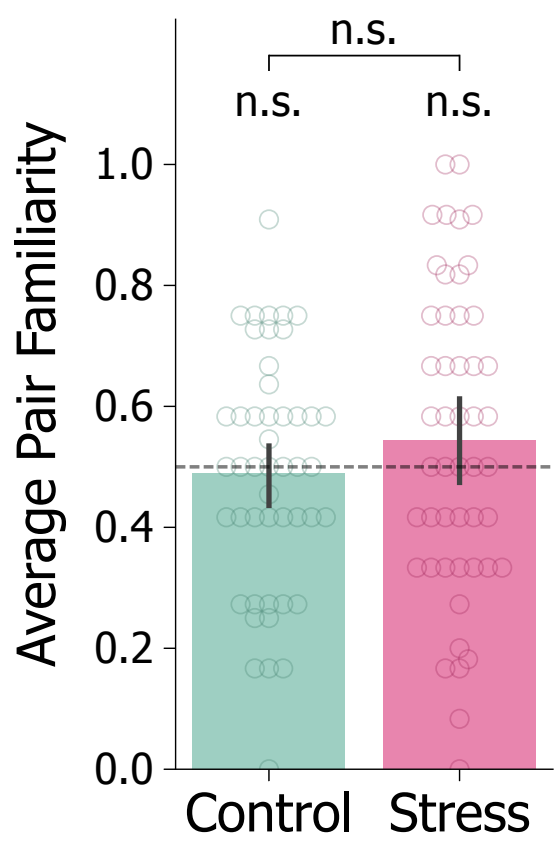

Figure S5. Behavioural performance on pair familiarity test. Mean accuracy is displayed for each group. Performance was not significantly above chance (0.5) in either the control (95% CI: $[0.43, 0.54], p = 0.359, r = 0.045$) or stress (95% CI: $[0.47, 0.61], p = 0.114, r = 0.196$) groups, and there was no significant difference between groups (95% CI: $[-0.036, 0.144], p = 0.237, r = 0.124$). Dots = individual participants. Error bars = bootstrapped 95% CIs.

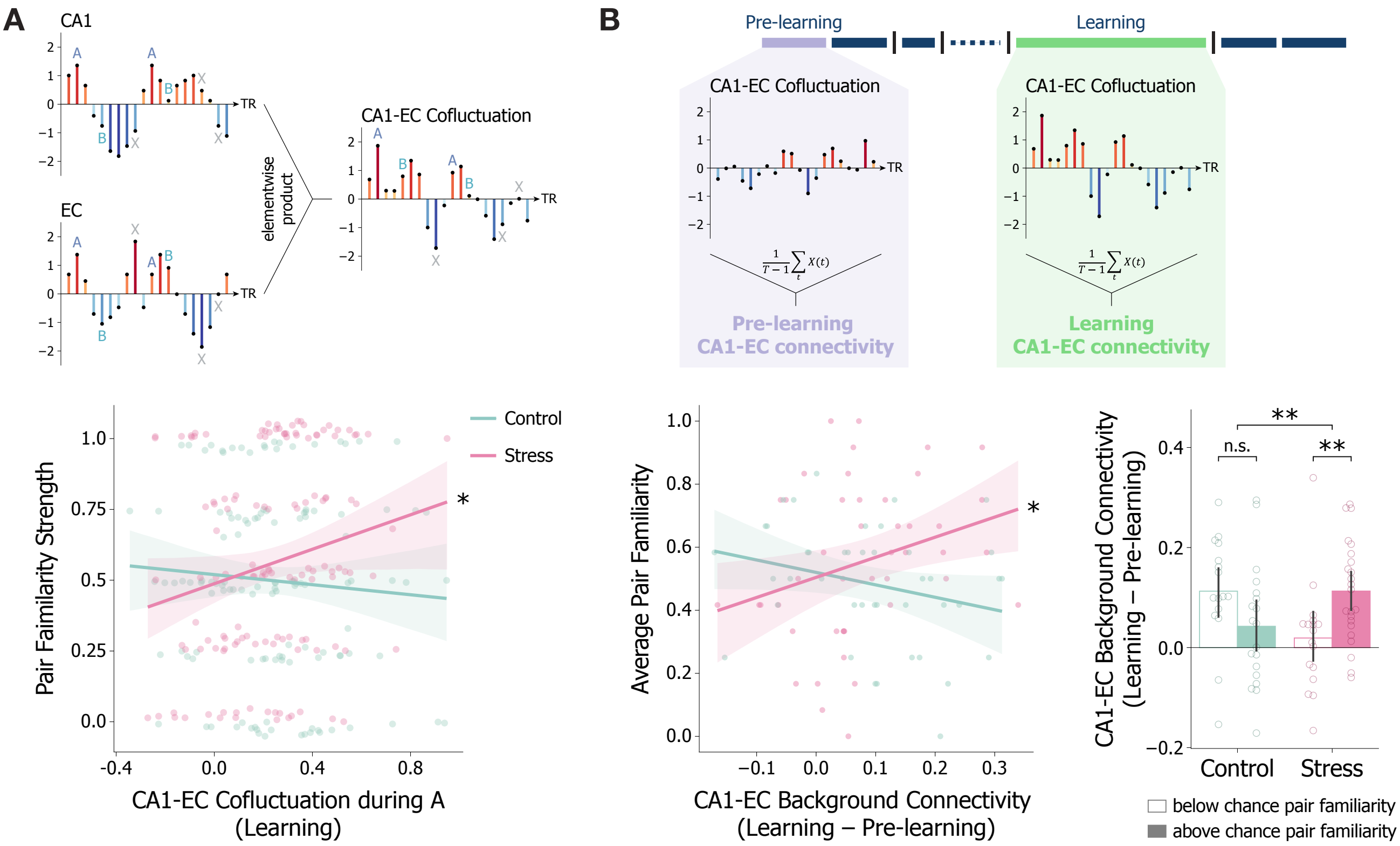

Figure S6. Relationships between CA1-EC connectivity during learning and performance on the pair familiarity task. **(A)** Momentary cofluctuation between CA1-EC is computed by taking the elementwise product of the z-scored mean timeseries from CA1 and EC during learning. Timepoints that occur during A images (offset by 3 TRs to account for haemodynamic lag) are used for analyses (top). The relationship between mean CA1-EC cofluctuation during each A category and pair familiarity strength for the corresponding category pair is shown for each group. Stronger CA1-EC cofluctuation was positively associated with pair familiarity strength in the stress group ($F(1, 89) = 4.90, p = 0.029, \eta_p^2 = 0.041$), but not in the control group ($F(1, 83) = 0.88, p = 0.35, \eta_p^2 = 0.007$; cofluctuation $\times$ group interaction ($F(1, 172) = 5.24, p = 0.023, \eta_p^2 = 0.022$). Dots = individual A-B pairs (3 per participant). Solid lines = predicted values from within-group LMEs. Error shading = bootstrapped 95% CIs over LME prediction values from 9,999 iterations of within-group participant resampling. Binary version of this analysis shown in main text Fig. 4B. **(B)** We employed a background connectivity approach to examine whether overall CA1-EC connectivity during learning is related to statistical learning at the participant level. As with the momentary cofluctuation analysis, we first used a GLM to regress out stimulus-evoked responses. We then extracted the mean of the model residuals across voxels in CA1 and EC. Background connectivity was finally calculated as the Pearson correlation of these mean time-series, normalized using a Fisher r-to-z transform. For each run, this value is equivalent to the mean of momentary cofluctuations across time (more specifically, the sum of the cofluctuation timeseries $X(t)$ divided by the total number of timepoints T minus 1) (top). The difference between CA1-EC background connectivity during the learning and pre-learning runs is positively associated with average pair familiarity performance in the stress group ($r(43) = 0.26, p = 0.023$), but not in the control group ($r(40) = -0.26, p = 0.068$). Pre-learning connectivity is subtracted from learning connectivity to isolate changes in connectivity elicited by statistical learning (left) With stress, participants with above-chance (> 0.5) performance on the pair familiarity test showed a significantly greater increase in CA1-EC background connectivity during learning compared to those with below-chance (< 0.5) performance (95% CI: $[0.027, 0.157], p = 0.008, r = 0.545$). This relationship was not apparent in the control group, which showed an opposite – though nonsignificant – effect (95% CI: $[-0.143, 0.006], p = 0.070, r = 0.401$); familiarity $\times$ group interaction ($F(1, 71) = 10.13, p = 0.002, \eta_p^2 = 0.125$) Participants with at-chance ($= 0.5$) performance on the pair familiarity test were not included in this binarized analysis (right). Dots = individual participants. Error shading = bootstrapped 95% CIs. *$p < 0.05$, **$p < 0.01$

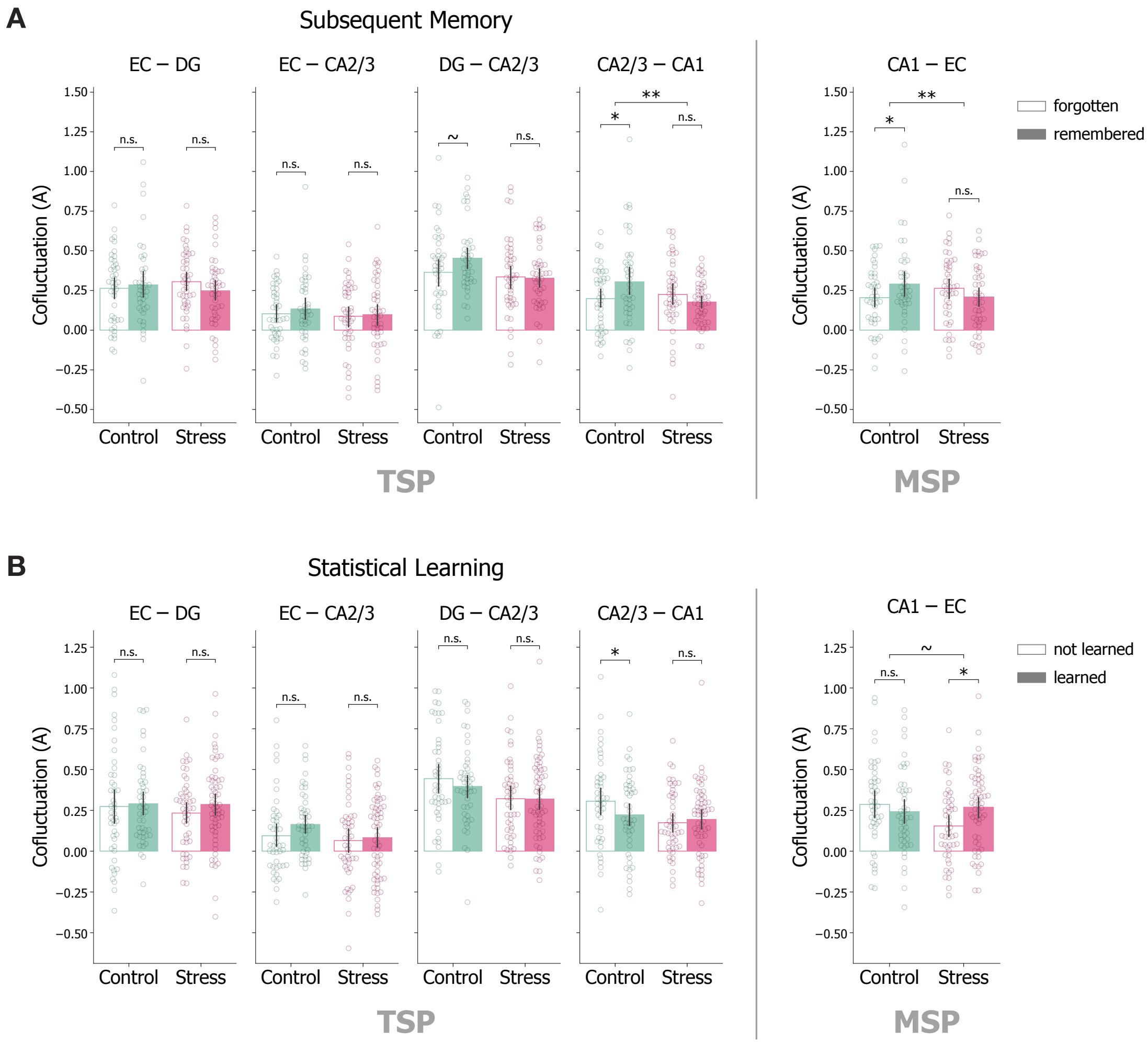

Figure S7. Momentary cofluctuation analyses along trisynaptic pathway connections. **(A)** Momentary cofluctuation between pairs of ROIs corresponding to TSP connections (EC-DG, EC-CA2/3, DG-CA2/3, CA2/3-CA1) for predictive A items that were subsequently remembered and forgotten within each group. For EC-DG and EC-CA2/3, cofluctuation was not significantly related to episodic memory in either the control (EC-DG: $F(1, 41) = 0.25, p = 0.617, \eta_p^2 = 0.004$; EC-CA2/3: $F(1, 41) = 0.53, p = 0.471, \eta_p^2 = 0.007$) or stress (EC-DG: $F(1, 44) = 1.96, p = 0.168, \eta_p^2 = 0.024$; EC-CA2/3: $F(1, 44) = 0.056, p = 0.813, \eta_p^2 = 0.001$) groups. DG-CA2/3 cofluctuation was marginally related to episodic memory in the control group, with higher cofluctuation for subsequently remembered compared to forgotten images ($F(1, 41) = 3.56, p = 0.066, \eta_p^2 = 0.051$), but there was no apparent association between DG-CA2/3 cofluctuation and episodic memory in the stress group ($F(1, 44) = 0.034, p = 0.855, \eta_p^2 = 0.000$; subsequent memory × group: $F(1, 85) = 2.19, p = 0.142, \eta_p^2 = 0.015$; main effect of group: $F(1, 85) = 4.12, p = 0.045, \eta_p^2 = 0.027$). CA2/3-CA1 cofluctuation was also associated with episodic memory in the control group, with significantly higher cofluctuation for subsequently remembered compared to forgotten images ($F(1, 41) = 6.47, p = 0.015, \eta_p^2 = 0.099$), but this relationship was again not evident in the stress group ($F(1, 44) = 1.62, p = 0.210, \eta_p^2 = 0.019$; subsequent memory × group: $F(1, 85) = 7.56, p = 0.007, \eta_p^2 = 0.053$). Given the positive associations between TSP cofluctuation (in particular, along TSP connections downstream of DG) and episodic memory, the lack of effect in the stress group suggests that stress may impair episodic memory processing along TSP. For comparison, CA1-EC results are shown on the right. Dots = individual participants. **(B)** Momentary cofluctuation along TSP connections for predictive A items belonging to pairs that are either learned or not learned within each group. For EC-DG, EC-CA2/3, and DG-CA2/3, cofluctuation was not significantly related to statistical learning in either the control (EC-DG: $F(1, 49) = 0.013, p = 0.909, \eta_p^2 = 0.000$; EC-CA2/3: $F(1, 49) = 2.25, p = 0.140, \eta_p^2 = 0.025$; DG-CA2/3: $F(1, 49) = 0.90, p = 0.346, \eta_p^2 = 0.010$) or stress (EC-DG: $F(1, 62) = 1.16, p = 0.286, \eta_p^2 = 0.011$; EC-CA2/3: $F(1, 62) = 0.004, p = 0.952, \eta_p^2 = 0.000$; DG-CA2/3: $F(1, 62) = 0.001, p = 0.982, \eta_p^2 = 0.000$) groups. CA2/3-CA1 cofluctuation is negatively associated with statistical learning in the control group, with lower cofluctuation for images belonging to learned pairs ($F(1, 49) = 4.44, p = 0.040, \eta_p^2 = 0.057$), while no relationship was evident in the stress group ($F(1, 62) = 0.23, p = 0.631, \eta_p^2 = 0.002$; familiarity × group: $F(1, 111) = 3.10, p = 0.081, \eta_p^2 = 0.018$; main effect of group: $F(1, 85) = 4.32, p = 0.041, \eta_p^2 = 0.025$). The lack of positive associations between TSP cofluctuation and statistical learning is consistent with the hypothesized role of TSP. For comparison, CA1-EC results are shown on the right. Dots = individual A-B pairs (3 per participant). Error bars = bootstrapped 95% CIs. **$p < 0.01$, *$p < 0.05$, ~$p < 0.07$
